# Supplementary material for: A fast ceramic mixed OH−/H+ ionic conductor for low temperature fuel cells
Source: Nat Commun. 2024 Jan 30;15:909. doi: 10.1038/s41467-024-45060-1 (PMC10827789; doi:10.1038/s41467-024-45060-1)
Supplement: Supplementary file 1 — Supplementary Information [file 41467_2024_45060_MOESM1_ESM.pdf]

Supplementary Information for

A fast ceramic mixed OH^-/H^+ ionic conductor for low temperature fuel cells

Peimiao Zou[1], Dinu Iuga[2], Sanliang Ling[3], Alex J. Brown[1], Shigang Chen[1], Mengfei Zhang[1], Yisong Han[2], A. Dominic Fortes[4], Christopher M. Howard[4] and Shanwen Tao[1,5*]

[1] School of Engineering, University of Warwick, Coventry CV4 7AL, UK.

[2] Department of Physics, University of Warwick, Coventry CV4 7AL, UK.

[3] Advanced Materials Research Group, Faculty of Engineering, University of Nottingham, Nottingham NG7 2RD, UK.

[4] ISIS Neutron and Muon Spallation Source, Rutherford Appleton Laboratory, Harwell Science and Innovation Campus, Chilton, Oxfordshire, OX11 0QX, UK.

[5] Department of Chemical Engineering, Monash University, Clayton, Victoria 3800, Australia.

Table of Contents

Supplementary Notes 1-5 3

Supplementary Figures 1-40 15

Supplementary Tables 1-9 57

Supplementary References 66

Supplementary Notes 1-5

Supplementary Note 1. Identification of the charge carrier in low temperature ionic conductors

The low temperature concentration cell technology was used to measure the charge carriers in perovskite oxide electrolyte in order to figure out which ions are moving in the materials[1].

The Ag/Ag_2O electrode was prepared through the electrochemical oxidation of sterling silver. Pieces of silver foil were sanded smooth with a fine silicon carbide sandpaper. Then, the silver foil was oxidized electrochemically in 1 mol L^{-1} of NaOH (98%, Alfa Aesar) at a current density of 1 mA cm^{-2} through a conventional three-electrode set-up which consisted of a silver foil, Pt mesh and Ag/AgCl (sat. KCl) electrode used as the working, counter, and reference electrode respectively. To avoid forming silver peroxide (AgO), the electrochemical oxidation process was stopped after the power voltage increasing to 0.6 V vs the standard hydrogen electrode (SHE),[2] i.e. 0.4 V vs Ag/AgCl reference electrode.

In a concentration cell of NaOH, the concentrations of Na^+ and OH^- were 10^{-2} M (pH=12) for the anode and 10^{-4} M (pH=10) for the cathode respectively. Ag/Ag_2O electrodes were sensitive to the concentration of OH^- because the following reactions occur on the electrodes[3]:

Anode: $2Ag + 2OH^- \rightarrow Ag_2O + H_2O + 2e^-$ pH=12 [OH]=10^{-2} M (1)

Cathode: $Ag_2O + H_2O + 2e^- \rightarrow 2Ag + 2OH^-$ pH=10 [OH]=10^{-4} M (2)

The concentration difference of Na^+ and OH^- on the two sides of the electrolyte membrane generated the membrane potential (ΔE). If the permeabilities of Na^+ and OH^- are equal (i.e.,

membrane potential (ΔE) is 0), the standard electromotive force (E^0) can be calculated by the Nernst's equation[4]:

$$E^0 = - RT/F \ln a(OH^-)_{Cathode} / a(OH^-)_{Anode} = - 0.059\times\log 10^{-4}/10^{-2} = 0.118 \text{ V} \quad (3)$$

The electromotive force (E) between the cathode and anode electrodes of concentration cell were measured by a Solartron 1470E CellTest System for at least 30 mins to record the voltage potential of plateau. The membrane potential (ΔE) can be determined using the electromotive force (E):

$$\Delta E = E - E^0 = E - 0.118 \text{ V} \quad (4)$$

If the electrolyte membrane shows cation conductivity, Na^+ moves from the anode side (high concentration) to the cathode side (low concentration), the cathode side of the membrane will become positively charged (ΔE >0). If the membrane shows anion conductivity, OH^- moves from the anode side to the cathode side, the cathode side of the membrane will become negatively charged (ΔE <0).

Hittorf's method was used to evaluate the permeabilities of Na^+ and OH^- as follows[5]:

$$\Delta E = - RT/F \left(t_{Na+} \ln \left(a(Na^+)_{Cathode} / a(Na^+)_{Anode}\right) - t_{OH-} \ln \left(a(OH^-)_{Cathode} / a(OH^-)_{Anode}\right)\right) \quad (5)$$

$$t_{Na+} + t_{OH-} = 1 \quad (6)$$

According to equations above, transport numbers of Na^+ (t_{Na+}) and of OH^- (t_{OH-}) are obtained. As shown in Supplementary Table 3, when a Nafion® membrane was used as the electrolyte of the cell, ΔE_{Nafion} was +0.077 V, indicating that Na^+ in the anode moved to the cathode side more easily than OH^- did, and Nafion® membrane shows a high t_{Na+} value of 0.87.

On the other hand, when an anion exchange membrane FAA was used as the electrolyte, ΔE_{FAA} was -0.104 V, indicating that OH^- in the anode moved to the cathode side more easily than Na^+ did. According to the calculation, FAA membrane shows a high t_{OH-} value of 0.93. The membrane potential (ΔE) of hydrated $SrZr_{0.8}Y_{0.2}O_{3-\delta}$ (SZYO20) shows negative value. Therefore, the main carriers in these perovskite oxides are main OH^- ions[4]. The transport number of OH^- for SZYO20 is about 0.7. Not only the main anion conductivity, but also the cation conductivity is presented. Although it is Na^+ used in the concentration cell to determine the transport number of cation, in this study, the available cations for perovskite oxide SZYO20 itself are only Sr^{2+}, Zr^{4+} and Y^{3+} which are too large to be conducted in the oxide. The likely mobile cations in hydrated SZYO20 are protons. This has been verified by the proton transference number (~0.27) of SZYO20 pellet at room temperature evaluated by chronoamperometry in conjunction with EIS measurements (Supplementary Fig. 11), which is close to the transport number of Na^+ for SZYO20 measured by concentration cells. Therefore, the hydrated SZYO20 is a mixed OH^-/H^+ conductor. This is also consistent with the solid state NMR results in which signals assigned to OH^- and to H^+ were detected in wet SZYO20 samples (Fig. 5a-d). The electronic conduction is very small according to measured electronic transfer number.

Supplementary Note 2. Raman spectra measurements and analyses

The second phase SrY_2O_4 hydrolysed when the SZYO20 sample was hydrated at 90 °C. The Raman features at 1071 cm^{-1} is related to $SrCO_3$[6], which is the product from hydrolysis of SrY_2O_4. The signal of $SrCO_3$ indicates the higher hydration level of small second phase SrY_2O_4 in SZYO20 samples (Supplementary Fig. 20a). It was reported that the hydroxyl groups formed at the ceramic surface can be detected in Raman spectra shown as narrow peaks at 3500-3650 cm^{-1}[7]. In Supplementary Fig. 20b, for SZYO20 samples with different hydration levels, the peak at 3620 cm^{-1} is stronger when the pellet is wetter. This peak is different from the typical peak at 3488 cm^{-1}[7] observed in the Raman spectra for commercial $Sr(OH)_2 \cdot 8H_2O$, indicating the hydroxyl groups formed on the perovskite oxide the SZYO20 are not from $Sr(OH)_2$. It is very likely to form hydrated metal oxyhydroxide, for example, $SrZr_{1-x}Y_xO_{3-\delta-y}(OH)_{2y} \cdot zH_2O$, where the upper limit for y is the oxygen deficiency δ.

The hydroxyl peak shifts to 3602 and 3532 cm^{-1} when A-site element was changed to Ba and Ca respectively (Supplementary Fig. 20c,d). After treatment in water, the solid samples appear to be substances with strong fluorescence, so the Raman peaks were not sharp. It is possible that the fluorescence annihilates the Raman peaks, especially after 3000 cm^{-1}.

The Raman peak at 726 cm^{-1} for SZYO20 samples shown in Supplementary Fig. 20f indicates that the Y doping, proton defects and oxygen vacancies perturbed the vibration of ZrO_6 octahedron in perovskite[6]. Part of Zr^{4+} in B-site of $SrZrO_3$ (SZO) is replaced by Y^{3+} upon yttrium doping forms negatively charged defects which must be compensated by creating extra oxygen vacancies[8]. The Raman spectra in the 600-900 cm^{-1} spectral region presents a peak shift of lattice vibrations in perovskite which is caused by the oxygen defects.[8]

Supplementary Note 3. Neutron powder diffraction analysis of SZYO20

Neutron powder diffraction (NPD) data of SZYO20 were collected on the time-of-flight high-resolution powder diffractometer (HRPD) at ISIS. The purpose of neutron diffraction was to identify the position of OH^- and/or hydrogen in the structure and more reliably determine oxygen positions. For Rietveld refinement data from detector bank 1, the high-resolution backscattering bank, were used.

For SZYO20 two samples were prepared, one vacuum dried to establish the anhydrous structure and one deuterated sample. For the dried sample of SZYO20 the data could be fit with two phases: $SrZr_{1-x}Y_xO_{3-\delta}$ and SrY_2O_4. The refinement process of the dried SZYO20 sample proceeded as follows: first cation atomic displacement parameters were refined, then Sr positions. This was followed by refinement of O positions, then O site occupancies were refined improving the goodness of fit. The O1 site was found to refine close to a value of unity, hence was fixed to one for the remainder of the refinement. The O2 site refined to an occupancy of 0.963(6), which gives an overall oxygen content of 2.93 within error i.e., δ = 0.07 consistent with result obtained from X-ray diffraction (XRD) in Supplementary Table 1. Note the mixed Zr/Y site occupancies were not refined as they have very similar neutron scattering lengths (Zr 7.16 fm, Y 7.75 fm)[9], so the relative Zr:Y ratio of perovskite phase was deduced from the quantity of oxygen vacancies. That is, the occupancies of Zr and Y were fixed to values of 0.86 and 0.14 respectively. The occupancy of the strontium site was refined to be close to one and was therefore set to one. The final Rietveld fit for the dried SZYO20 model against data from bank 1 is shown in Supplementary Fig. 25a, the corresponding structural parameters are shown in Supplementary Table 6.

For the deuterated SZYO20 sample the anhydrous structure was used to give reasonable initial fits, and the final Rietveld fit is shown in Supplementary Fig. 25b and Supplementary Table 7. Fourier difference F_{obs}- F_{calc} maps were then generated to attempt to locate the position of deuteron in the structure. A small anomaly in the Fourier maps was observed adjacent the O2 oxygen which was then used as an initial position to insert and refine deuteron. Given the low D occupancy and the possibility of O-H(D) disorder we cannot be certain that the deuterated structure shown in Supplementary Fig. 25c is correct, but several pieces of information give us confidence. Firstly, the refined D position corresponds to a small anomaly observed in the Fourier difference maps. Secondly, the fit improved with the introduction of D and the occupancy refined to reasonable values based on the expected quantity of vacancies in the structure. Thirdly, the O-D lengths are chemically reasonable and consistent with previous results. The distances between hydrogen and an O2 nearest-neighbour are 2.3054(5) Å and 2.1925(5) Å (Supplementary Fig. 25c), while O2-O2 distances are 2.99346(6) Å. A combination of intra-proton transfer and OH^- migration between these sites could be taking place resulting in the high conductivity observed in this material.

Supplementary Note 4. Calculation of relative density of perovskite oxide pellets

To calculate the bulk crystal density (D) of a unit cell, the equation is expressed as follows:

$$D = \frac{FW \times Z \times 1.66}{V} \quad (7)$$

Where FW is formula weight of the perovskite oxide with the unit of g mol^{-1}, V is volume of the unit cell with the unit of Å^3, which is obtained from Rietveld refinement, Z is the number of formula units in a unit cell, Z = 4 for $AZr_{1-x}Y_xO_{3-\delta}$ (A= Ca, Sr, x= 0, 0.1, 0.2), Z = 1 for $BaZrO_3$ (BZO) and $BaZr_{0.8}Y_{0.2}O_{3-\delta}$ (BZYO20). The unit for D is g cm^{-3}.

To calculate the real density (d) of a perovskite oxide pellet, the equation is expressed as follows:

$$d = \frac{m}{\lambda \times A} \quad (8)$$

Where m is mass, λ is thickness and A is cross-sectional area of the perovskite oxide pellet. The unit for d is g cm^{-3}.

In order to eliminate the error in dimension measurement, the real density (d) of a pellet can be also determined by Archimedes' principle, calculated as follows[10]:

$$d = m_{\text{in air}} \frac{\rho_{\text{liquid}}}{m_{\text{in air}} - m_{\text{in liquid}}} \quad (9)$$

Where ρ_{liquid} is the density of the liquid generating buoyancy, i.e., water in this work, $m_{\text{in air}}$ is the mass of the pellet in the air and $m_{\text{in liquid}}$ is the mass of the pellet in liquid.

The relative density (RD) is calculated as follows:

$$RD = \frac{d}{D} \times 100\% \tag{10}$$

Summary of the relative densities of oxide pellets are listed in Supplementary Table 9.

Supplementary Note 5. Electrolysis of $H_2{}^{18}O$ and D_2O using dense $CaZr_{0.8}Y_{0.2}O_{3-\delta}$ as the electrolyte

Electrolysis experiments were carried out to trace the OH^- conduction in an electrolytic cell.

To identify the transfer of oxygen in OH^- species during electrolysis, ^{18}O labelled water worked as a tracer in the experiment.

For electrolysis of $H_2{}^{18}O$, the hydrogen revolution reaction (HER) happens on the cathode of electrolyser is:

$$2\ H_2{}^{18}O + 2\ e^- \rightarrow 2\ {}^{18}OH^- + \frac{1}{2}\ H_2 \quad (11)$$

The oxygen revolution reaction (OER) happens on the anode of electrolyser is:

$$2\ {}^{18}OH^- \rightarrow H_2{}^{18}O + \frac{1}{2}\ {}^{18}O_2 + 2e^- \quad (12)$$

The isotopic water (20% $H_2{}^{18}O$ in $H_2{}^{16}O$) was pumped into the cathode chamber to work as the reactant of HER, while normal deionized water $H_2{}^{16}O$ was pumped into the anodic chamber to keep the electrolyte pellet moist. If the OH^- ions are conducted through the dense $CaZr_{0.8}Y_{0.2}O_{3-\delta}$ (CZYO20) pellet, under certain voltage for a long time, some of $^{18}OH^-$ ions will transfer from cathode to anode to produce $H_2{}^{18}O$ species in OER process, thus the ^{18}O-labelled species like $H_2{}^{18}O$ or $^{18}OH^-$ should be detected in the OER side of the cell.

The $H_2{}^{18}O$ and $H_2{}^{16}O$ species are hard to directly distinguish by Raman and Fourier-transform infrared spectra (FTIR) because the isotopic shift for ^{18}O-H and ^{16}O-H stretching vibrations is barely noticeable especially when the ^{18}O content is very low. However, it has been reported that when NaOCl diluted in $H_2{}^{18}O$ was employed, a new band at 684 cm^{-1} along with

the O-Cl stretching band at 711 cm^{-1} was observed in Raman spectra.[11] Therefore, the aqueous solution of OER side during $H_2{}^{18}O$ electrolysis was sampled at regular intervals with NaOCl as an additive for Raman measurement. ^{18}O-labelled NaOCl was prepared by mixing 1 mL NaOCl (15% in $H_2{}^{16}O$) with 3 mL of ^{18}O labelling samples of OER side. In Supplementary Fig. 36b, for sample 20% $H_2{}^{18}O$ with NaOCl additive, the observed Raman shift at 711 cm^{-1} and 683 cm^{-1} was assigned to ^{16}O-Cl and ^{18}O-Cl stretch respectively, which agrees with the calculated shift based on the two atom approximation of O-Cl (28 cm^{-1})[11].

It was found the ^{18}O-Cl stretch signal became slightly stronger with extended duration of electrolysis as shown in Supplementary Fig. 36c,d, indicating $H_2{}^{18}O$ can continuously diffuse through the dense CZYO20 pellet under a certain applied DC voltage even though the current density is not high. When no voltage potential is applied on the electrolytic cell, the solution of the OER side was also sampled for comparison, the Raman spectra of which could not be fitted to the peak at 683 cm^{-1} (Supplementary Fig. 36e), indicating the ^{18}O labelling species could only diffuse through the solid electrolyte under a certain applied DC voltage, rather than spontaneously.

To further verify the transfer of OH^- species during electrolysis, deuterium in D_2O worked as a tracer, from which the vibration of the D-O bond can be detected in FTIR.

In this case, the HER happens on the cathode of electrolyser is:

$$2D_2O + 2e^- \rightarrow 2OD^- + \frac{1}{2}D_2 \quad (13)$$

The OER happens on the anode of electrolyser is:

$$2OD^- \rightarrow D_2O + \frac{1}{2}O_2 + 2e^- \quad (14)$$

or

$$OD^- + OH^- \rightarrow HDO + \frac{1}{2}O_2 + 2e^- \quad (15)$$

Similarly, the pure D_2O or solution of 1 M KOH dissolved in D_2O was pumped into the cathode chamber to work as the reactant of HER while pure H_2O water into the anodic chamber. If the OH^-/OD^- ions are conducted through the dense CZYO20 pellet, under certain voltage, the OH^-/OD^- ions will transfer from cathode to anode to produce D_2O or HDO species in OER process. Therefore, the aqueous solution of OER side during electrolysis experiment was sampling for FTIR measurement. Fresh H_2O, D_2O, and 1 M KOH dissolved in H_2O and D_2O were also measured by FTIR to identify the IR Spectra for H_2O and D_2O mixtures. CO_2 gas was purged into solutions to identify the existence of KOH.

When no voltage potential was applied on the electrolytic cell, the solution of OER side was also sampled for FTIR measurement, the results indicate neither D_2O nor KOH can diffuse through the dense CZYO20 pellet (Supplementary Figs. 37,38).

The diffusion of smaller H_2O is expected to be easier than D_2O, so the current density of $H_2{}^{18}O$ electrolysis is slightly higher than it of D_2O electrolysis (Supplementary Figs. 36a,37a), which is in an agreement with the conductive ability of this kind of solid electrolyte in H_2O and D_2O. As the KOH added in D_2O can significantly accelerate the HER reaction on cathode and improve the conductivity of CZYO20 pellet, the current density of KOH-D_2O electrolysis is much higher as shown in Supplementary Figs. 38a. The chemical and structure stability of the CZYO20 pellet is good (Supplementary Figs 38b,39,40) indicating a robust electrolyte had been used in the electrolytic cell. When 2 V DC voltage was applied through the KOH-D_2O

electrolytic cell for over 16 hours, the signal of HDO was observed in pure water on other side while no K_2CO_3 thus no KOH was observed indicating OD^- ions diffused from the KOH-D_2O side to the H_2O side while KOH was not (Supplementary Figure 38d). This experiment indicates the OD^- ions were transferred through the oxide materials, i.e., OH^- can be conducted in oxide materials.

Supplementary Figures 1-40

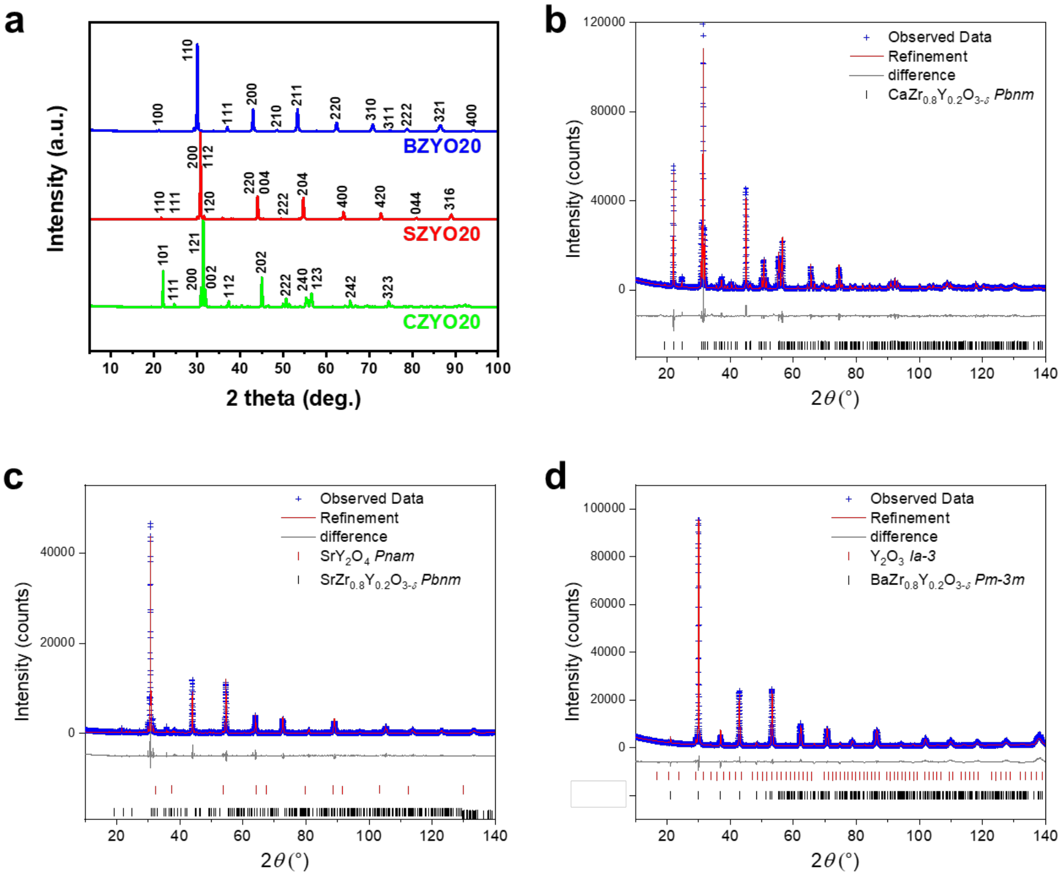

Supplementary Figure 1. XRD patterns of perovskite oxides synthesized by a combustion method. a, The XRD patterns of $AZr_{0.8}Y_{0.2}O_{3-\delta}$ (where A = Ca, Sr, Ba, denoted as CZYO20, SZYO20, BZYO20). **b-d**, Experimental (crosses), calculated (solid red line), and difference (grey line) results of XRD refinement for (**b**) CZYO20 (Black *hkl* ticks $CaZr_{0.8}Y_{0.2}O_{3-\delta}$), (**c**) SZYO20 (92% $SrZr_{0.8}Y_{0.2}O_{3-\delta}$ and 8% SrY_2O_4. Black *hkl* ticks $SrZr_{0.8}Y_{0.2}O_{3-\delta}$; red *hkl* ticks SrY_2O_4), (**d**) BZYO20 (99% $BaZr_{0.8}Y_{0.2}O_{3-\delta}$ and 1% Y_2O_3. Black *hkl* ticks $BaZr_{0.8}Y_{0.2}O_{3-\delta}$; red *hkl* ticks Y_2O_3).

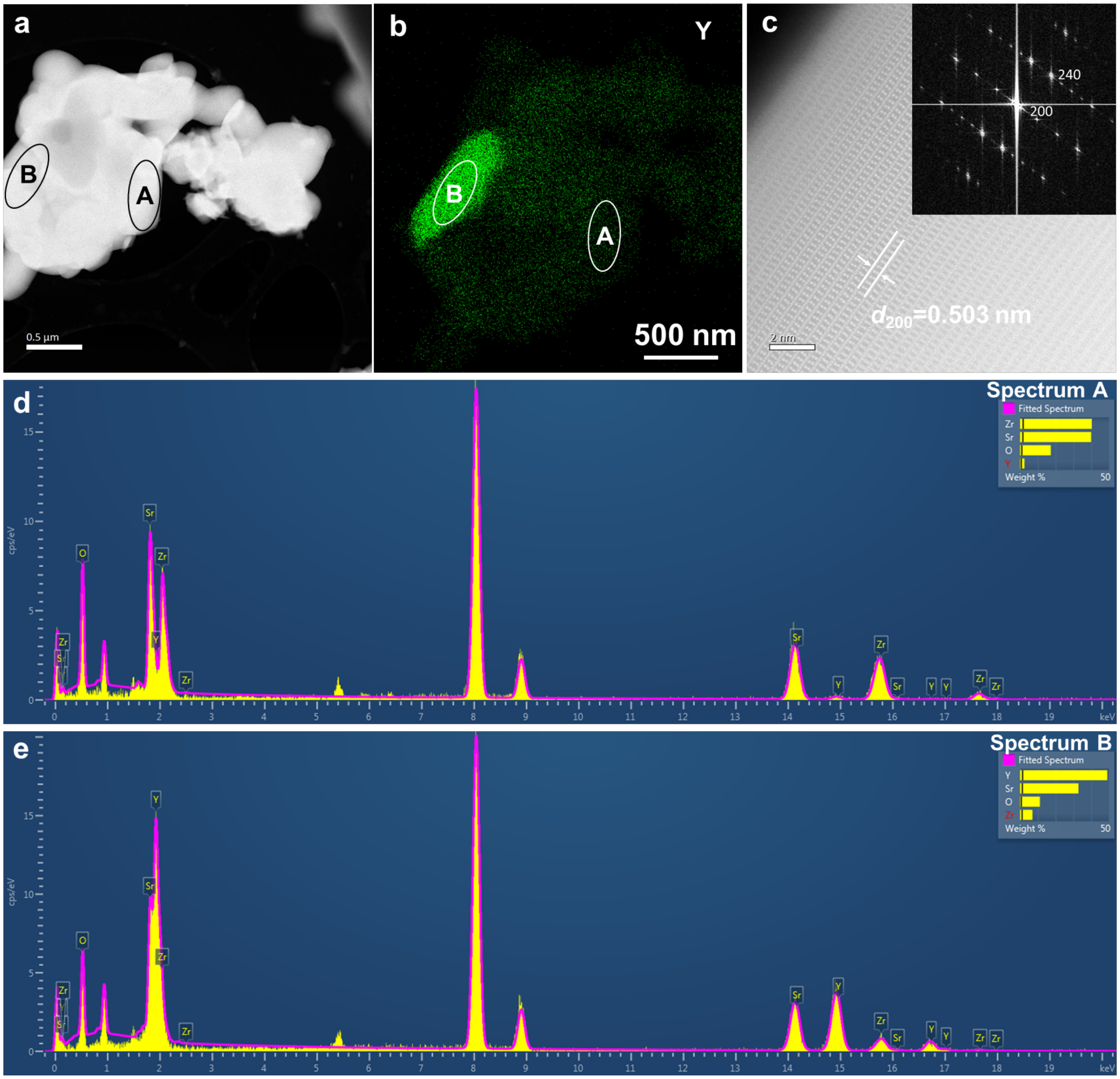

Supplementary Figure 2. STEM and EDX analysis of SZYO20 powders. a, ADF-STEM image. **b**, EDX map of Y, indicating area B is Y-rich. **c**, High resolution ADF-STEM image taken from area B in **a** (inset: corresponding FFT of the image). **d**,**e**, EDX spectra extracted from area A (**d**) and B (**e**) in **b**, respectively.

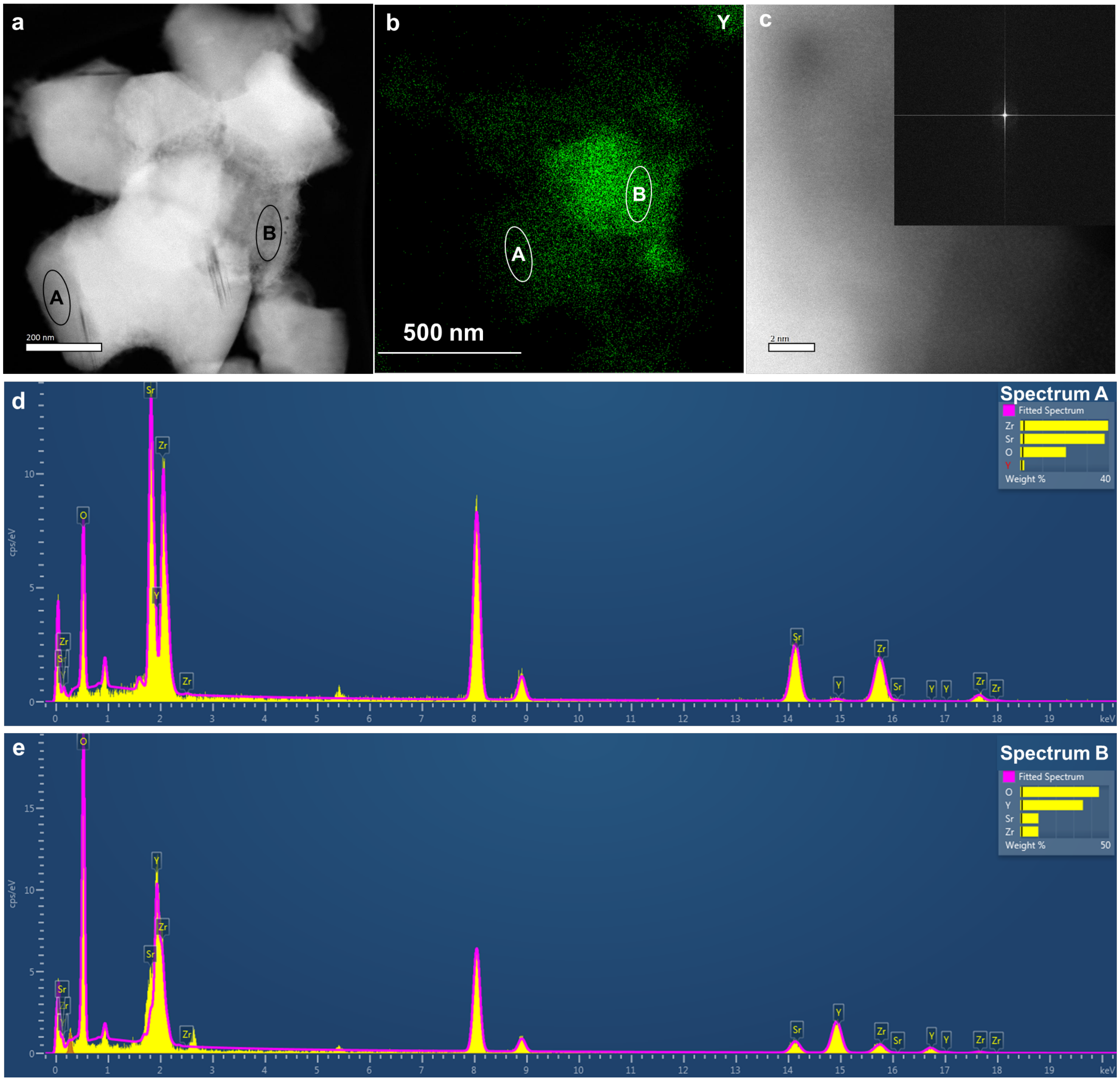

Supplementary Figure 3. STEM and EDX analysis of washed SZYO20 powders. a, ADF-STEM image. **b**, EDX map of Y, indicating area B is Y-rich. **c**, High resolution ADF-STEM image taken from area B in **a** (inset: corresponding FFT of the image). **d**,**e**, EDX spectra extracted from area A (**d**) and B (**e**) in **b**, respectively.

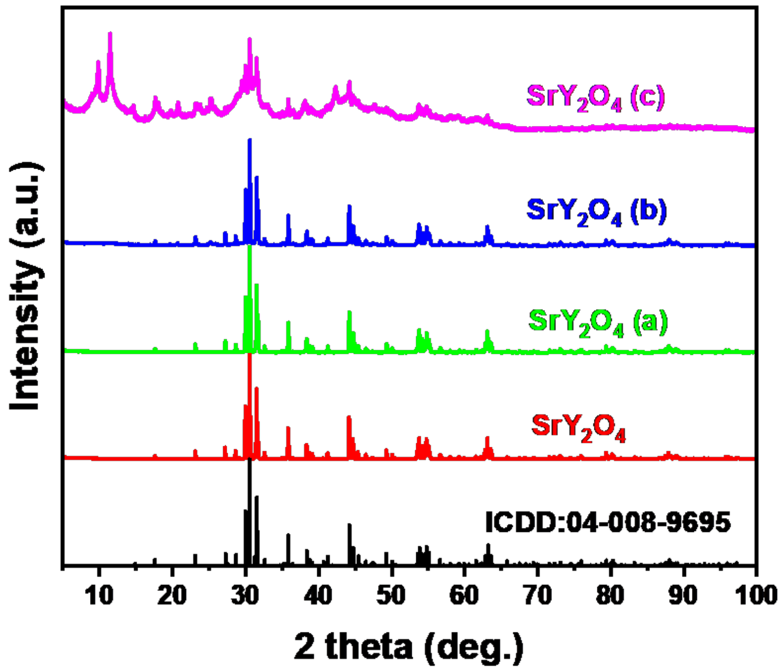

Supplementary Figure 4. The XRD patterns of synthesized SrY_2O_4 and sample after immersed in water (a) at room temperature for 4 days, (b) heated to 90 °C for 1 hour and (c) heated to 90 °C for over 10 hours.

5

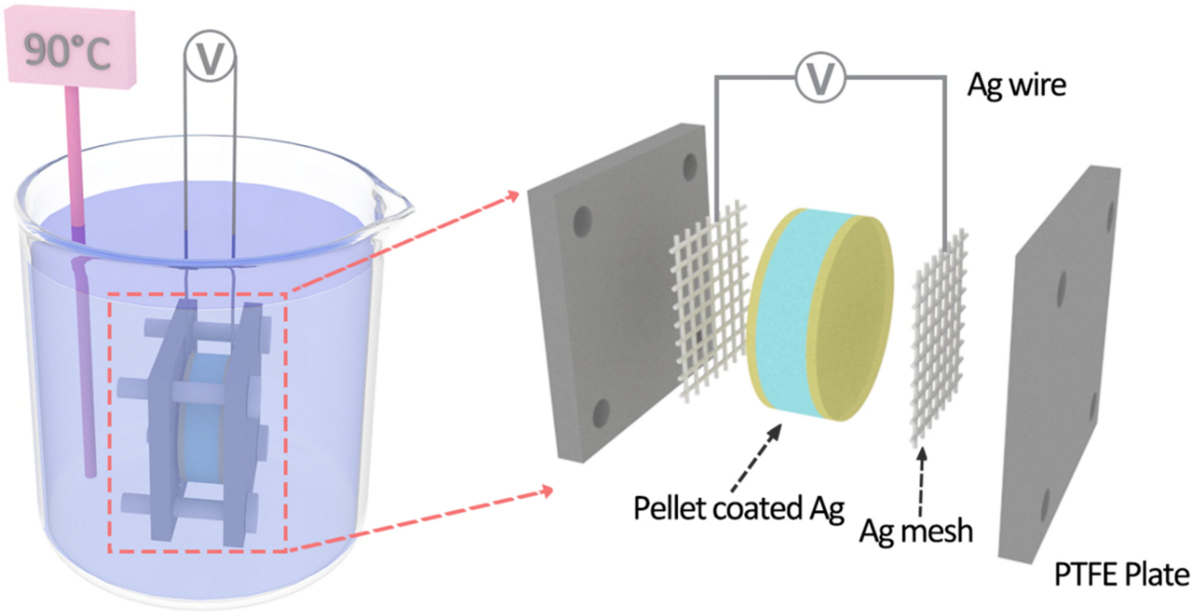

Supplementary Figure 5. Schematic diagram of the home-made set-up for conductivity measurements in water.

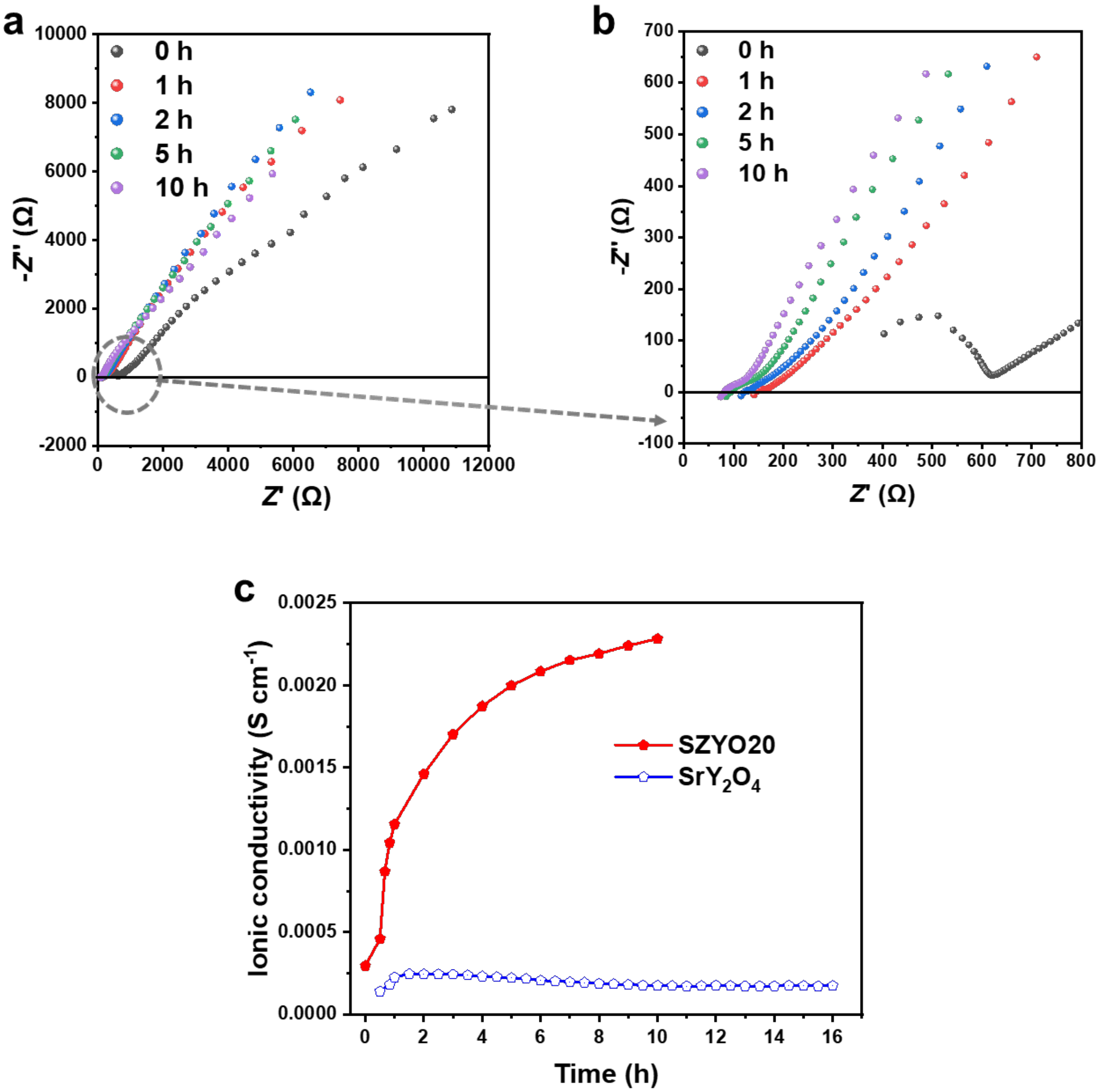

Supplementary Figure 6. The conductivity of un-washed SZYO20 pellet and the second phase SrY_2O_4. a,**b**, The (**a**) representative a.c. impedance of sample SZYO20 in water at room temperature at 0, 1, 2, 5 and 10 hours, and (**b**) enlarged impedance spectra. **c**, The conductivity changes against time of sample SZYO20 and SrY_2O_4 in water at room temperature.

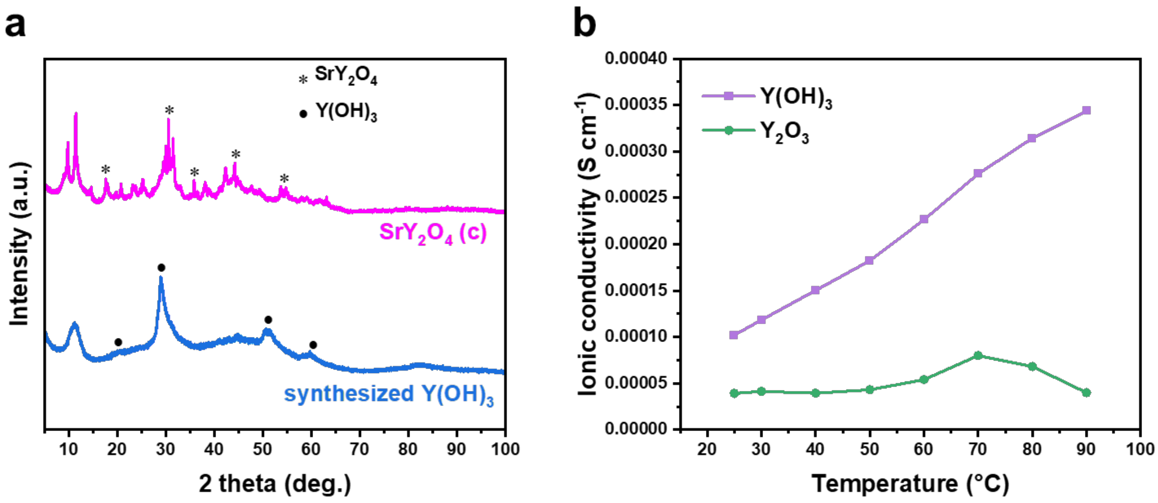

Supplementary Figure 7. The effect of the hydrolysis of second phase SrY_2O_4 on conductivity. a, The XRD patterns of hydrolyzed SrY_2O_4 and synthesized amorphous $Y(OH)_3$. **b**, The ionic conductivity of synthesized amorphous $Y(OH)_3$ and commercial Y_2O_3 pellets. The Y-rich area in washed SZYO20 is amorphous (Supplementary Fig. 3a-c). In order to rule out the contribution of insoluble residual products due to hydrolysis of SrY_2O_4 on the conductivity measurement, the poorly crystallized $Y(OH)_3$ was prepared. XRD pattern of synthesized $Y(OH)_3$ (Supplementary Fig. 7a) shows a bumped background, which reveals the presence of large amount of amorphous phase, and the diffraction peaks are consist with that of a monoclinic lattice of $Y(OH)_3$ (JCPDS No. 21-1447).[12] XRD patterns have shown that the as-prepared $Y(OH)_3$ was poorly crystallized, as same as the cathodic electrodeposited yttrium hydroxide in a literature.[13]

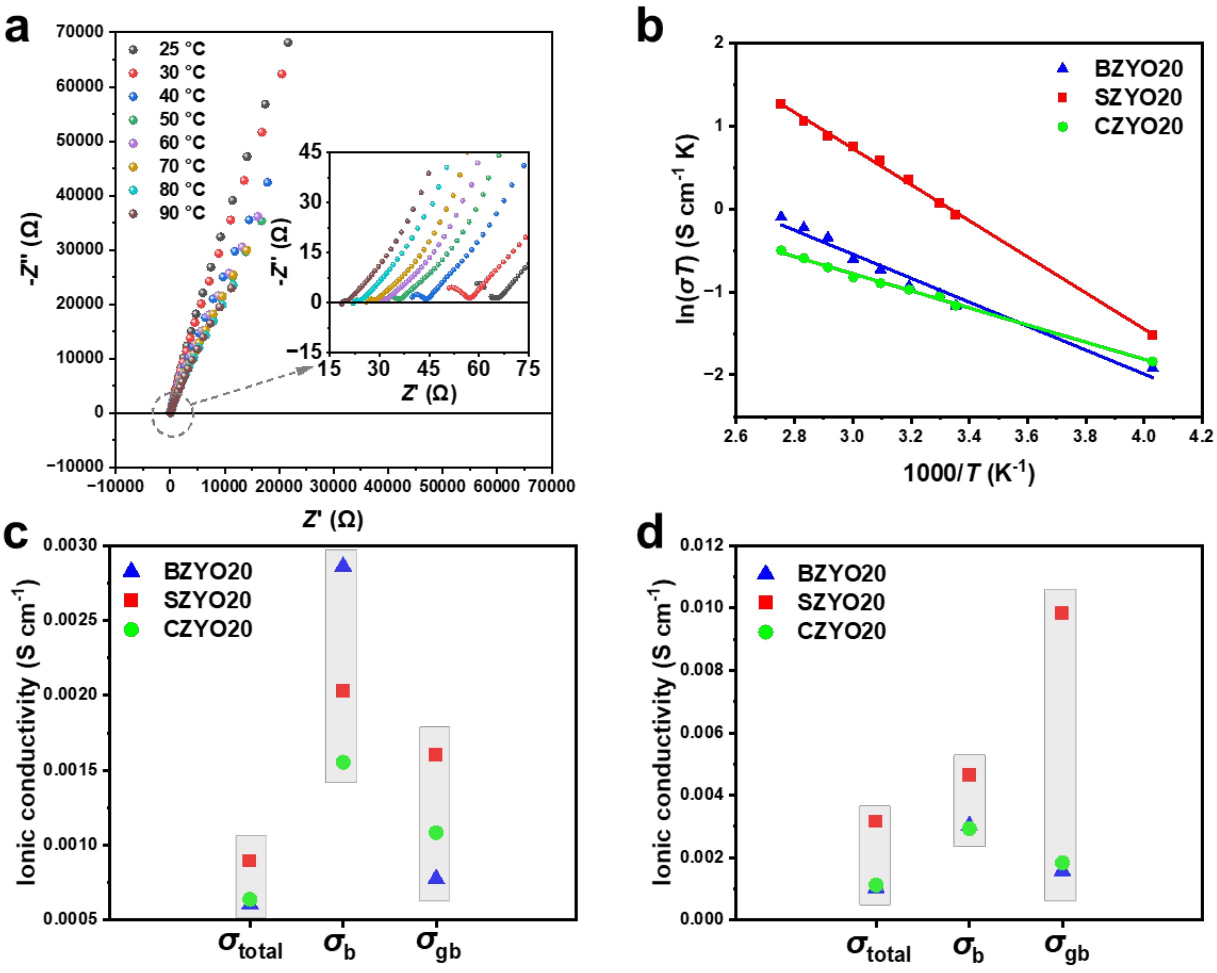

Supplementary Figure 8. Representative impedance and conductivity plots measured in the high-frequency (10 MHz) to low-frequency (10 mHz) range. a, Experimental impedance curves of SZYO20. Enlarged impedance spectra is displayed in insert. **b**, Arrhenius plots of conductivities of $AZr_{0.8}Y_{0.2}O_{3-\delta}$ where A = Ca, Sr, Ba. Measurements were carried out at -25 °C and 25 to 90 °C in water. The calculated activation energy E_a for BZYO20 is 0.15 eV, for SZYO20 is 0.18 eV, for CZYO20 is 0.09 eV. **c**,**d**, Total conductivity σ_{total} extracted to bulk conductivity σ_b and grain boundary conductivity σ_{gb} at (**c**) -25 °C and (**d**) 25 °C.

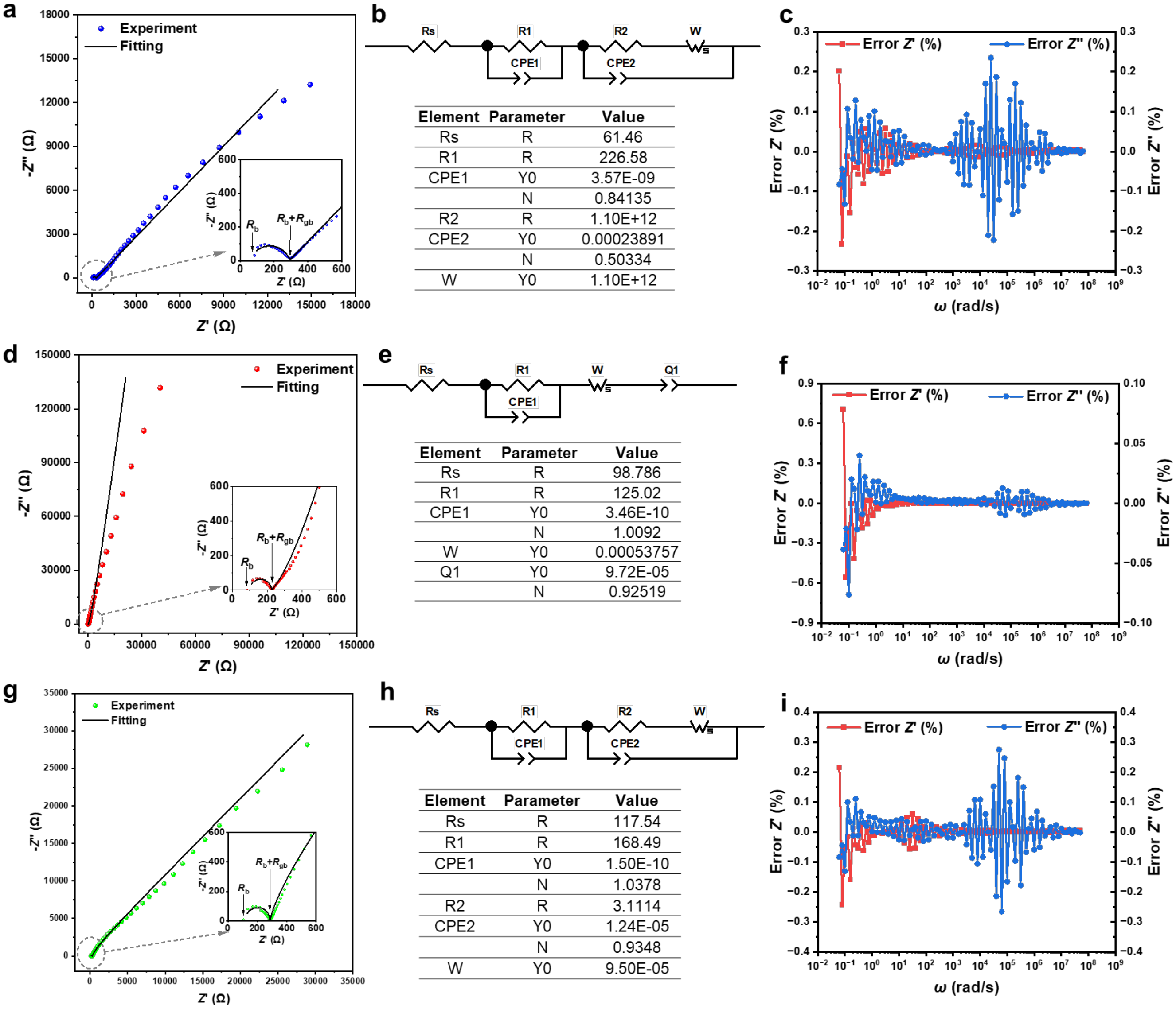

Element	Parameter	Value
Rs	R	61.46
R1	R	226.58
CPE1	Y0	3.57E-09
	N	0.84135
R2	R	1.10E+12
CPE2	Y0	0.00023891
	N	0.50334
W	Y0	1.10E+12

Element	Parameter	Value
Rs	R	98.786
R1	R	125.02
CPE1	Y0	3.46E-10
	N	1.0092
W	Y0	0.00053757
Q1	Y0	9.72E-05
	N	0.92519

Element	Parameter	Value
Rs	R	117.54
R1	R	168.49
CPE1	Y0	1.50E-10
	N	1.0378
R2	R	3.1114
CPE2	Y0	1.24E-05
	N	0.9348
W	Y0	9.50E-05

Supplementary Figure 9. Representative circuit fit and simulation results for impedance plots. a-c, For BZYO20 at -25 °C. (**a**) Fitted impedance curve. Enlarged impedance spectra is displayed in insert; (**b**) Equivalent circuit and parameters; (**c**) Results of the Kramers–Kronig validation test. **d-f**, For SZYO20 at -25 °C. **g-i**, For CZYO20 at -25 °C. The bulk R_b and grain boundary R_{gb} resistances have been extracted in (**a**), (**d**) and (**g**) due to the observation of grain boundary response with the estimated capacitance in 10^{-11}-10^{-8} farads obtained.[14] For the

equivalent circuit in (**b**), (**e**) and (**h**), R_s represents bulk ionic resistance, R_1 and CPE_1 represent the resistance and constant phase element (*CPE*) for grain boundary response. *W* is the Warburg impedance related to ion diffusion on the electrode-electrolyte interfaces, which is most prominent at low frequencies. R_2 and CPE_2 in (**b**) and (**h**) represent the sample-electrode interface response or electrode behaviours. Q_1 in (**e**) is also a constant phase element, which corresponds to the diffusion limitations in the electrode.

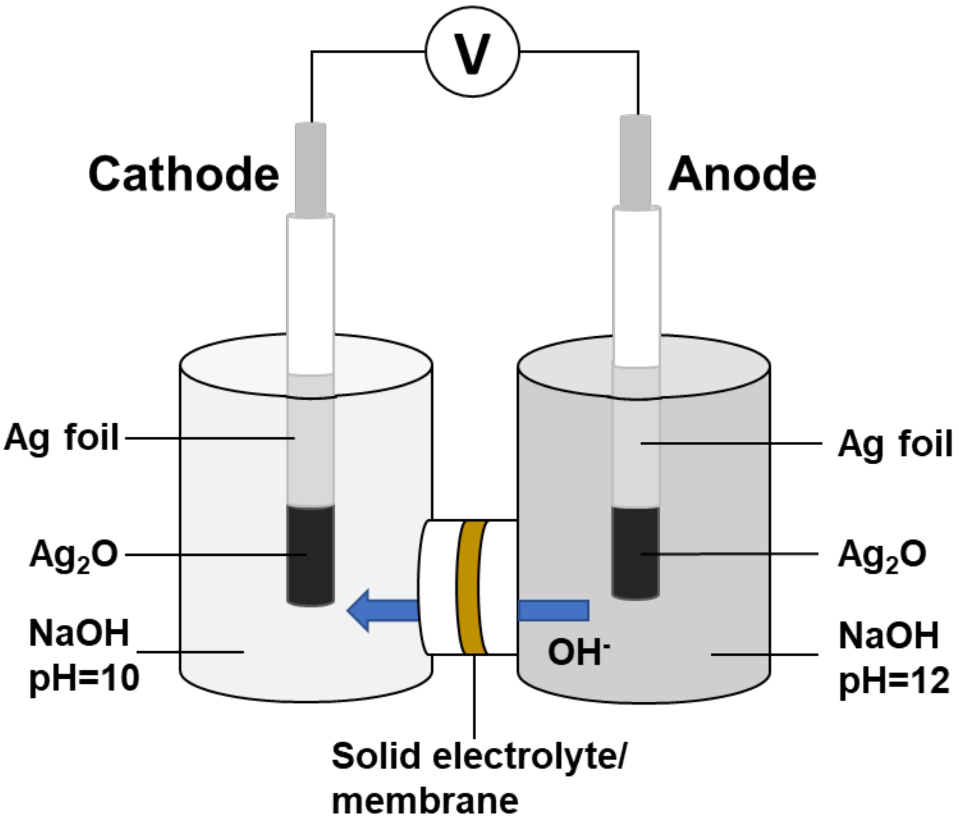

Supplementary Figure 10. Schematic diagram of the concentration cell to measure electrolyte membrane potential.

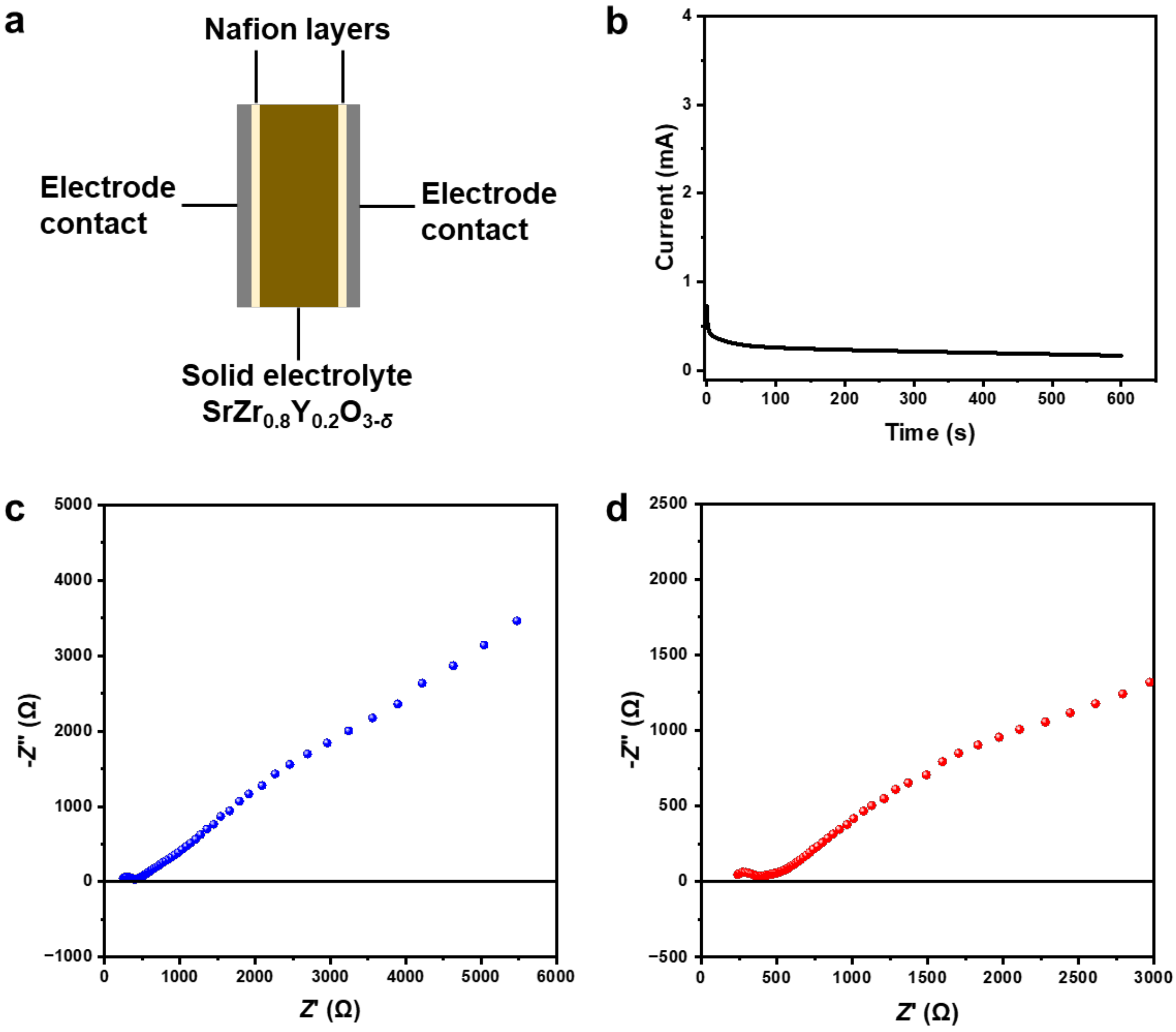

Supplementary Figure 11. Measurement of proton transference number of SZYO20 at room temperature. a, Schematic of Nafion|SZYO20 pellet|Nafion electrolyte assembly, which was measured in the set-up shown in Supplementary Fig.33. **b**, Polarization plot measured by chronoamperometry with applied DC voltage of 1 V. **c**,**d**, The a.c. impedance spectra measured (**c**) before and (**d**) after the polarization process.

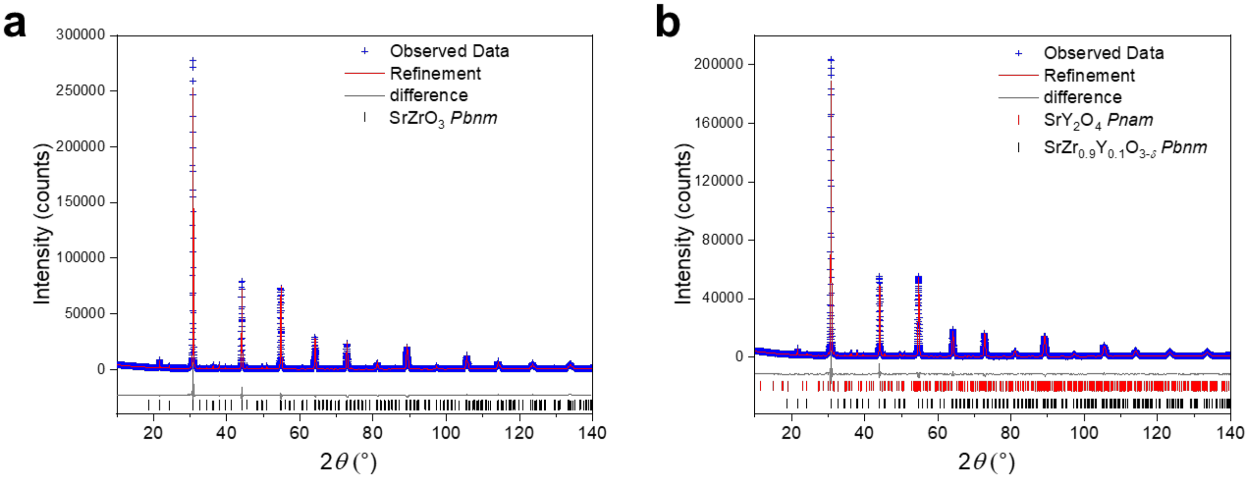

Supplementary Figure 12. Experimental (crosses), calculated (solid red line), and difference (grey line) results of XRD refinement. **a**, For $SrZrO_3$ (SZO) prepared by a combustion method. **b**, For $SrZr_{0.9}Y_{0.1}O_{3-\delta}$ (SZYO10) prepared by a combustion method. 98% $SrZr_{0.9}Y_{0.1}O_{3-\delta}$ and 2% SrY_2O_4.

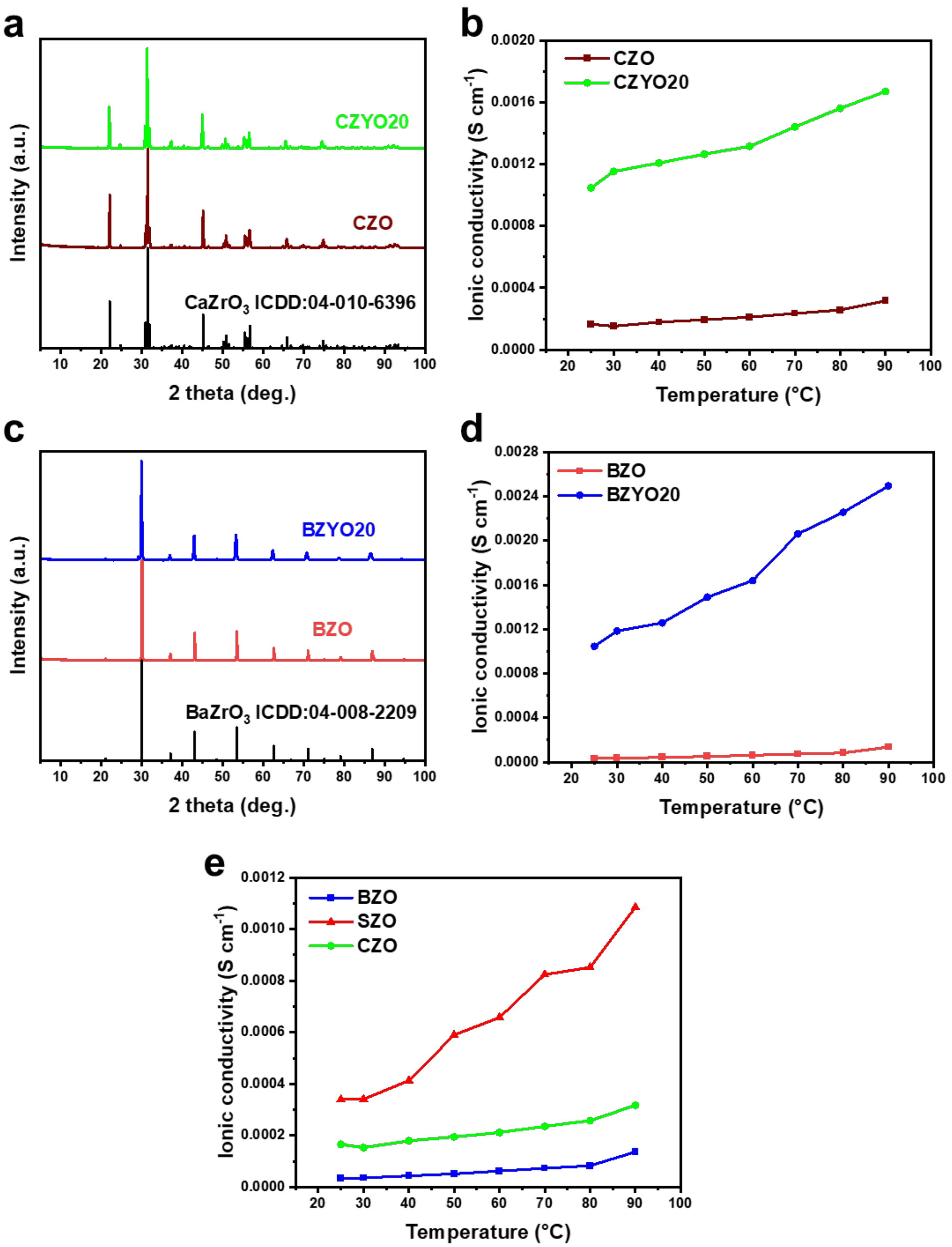

Supplementary Figure 13. The ionic conductivity of the parent zirconates. a,**b**, The (**a**) XRD patterns and (**b**) ionic conductivity of $CaZrO_3$ (CZO) and CZYO20. **c**,**d**, The (**c**) XRD patterns and (**d**) ionic conductivity of BZO and BZYO20. **e**, The ionic conductivity of $AZrO_3$ (A = Ca, Sr, Ba) in water.

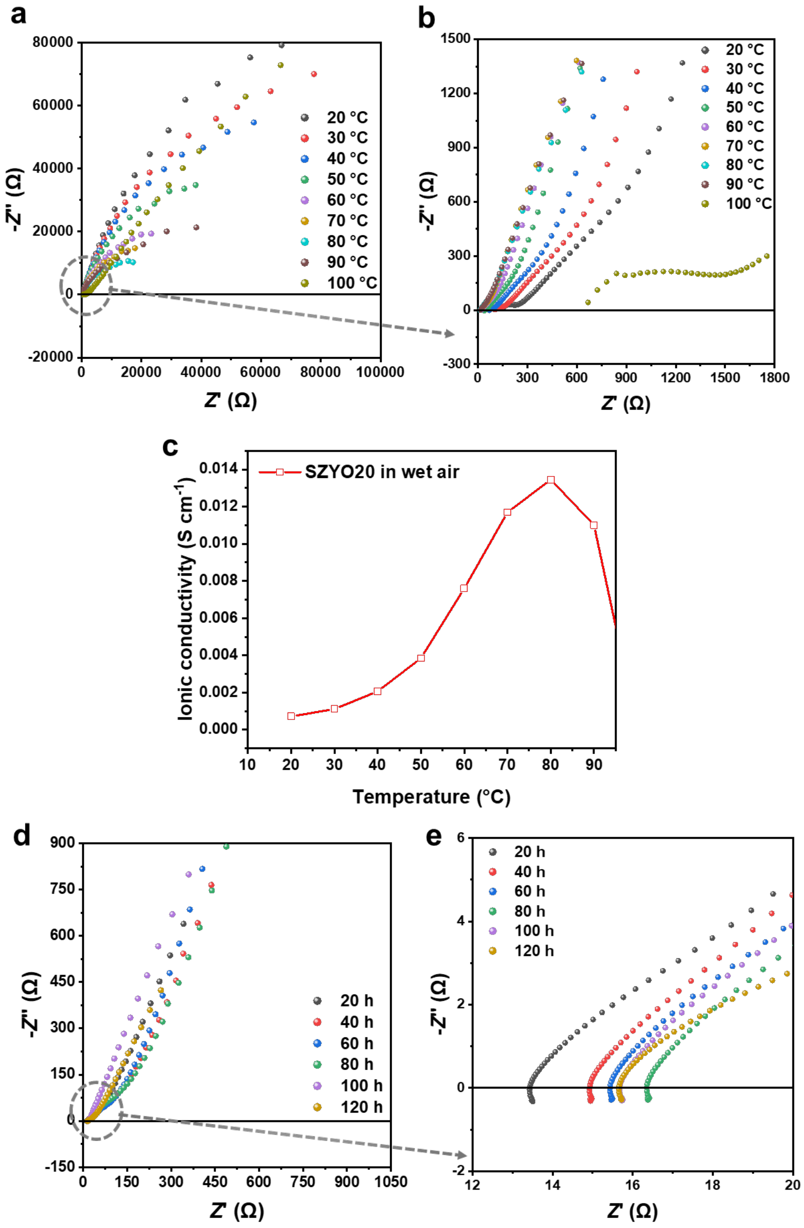

Supplementary Figure 14. Conductivity of SZYO20 in wet air. a,**b**, The (**a**) representative a.c. impedance spectra of SZYO20 in wet air and (**b**) enlarged impedance spectra. **c**, The conductivity of SZYO20 in wet air, enlarged plot between 20 and 90 °C associated with Fig. 4c. **d**,**e**, The (**d**) representative a.c. impedance spectra of SZYO20 in wet air at 70 °C at 20, 40, 60, 80, 100 and 120 hours associated with Fig. 4d, and (**e**) enlarged impedance spectra.

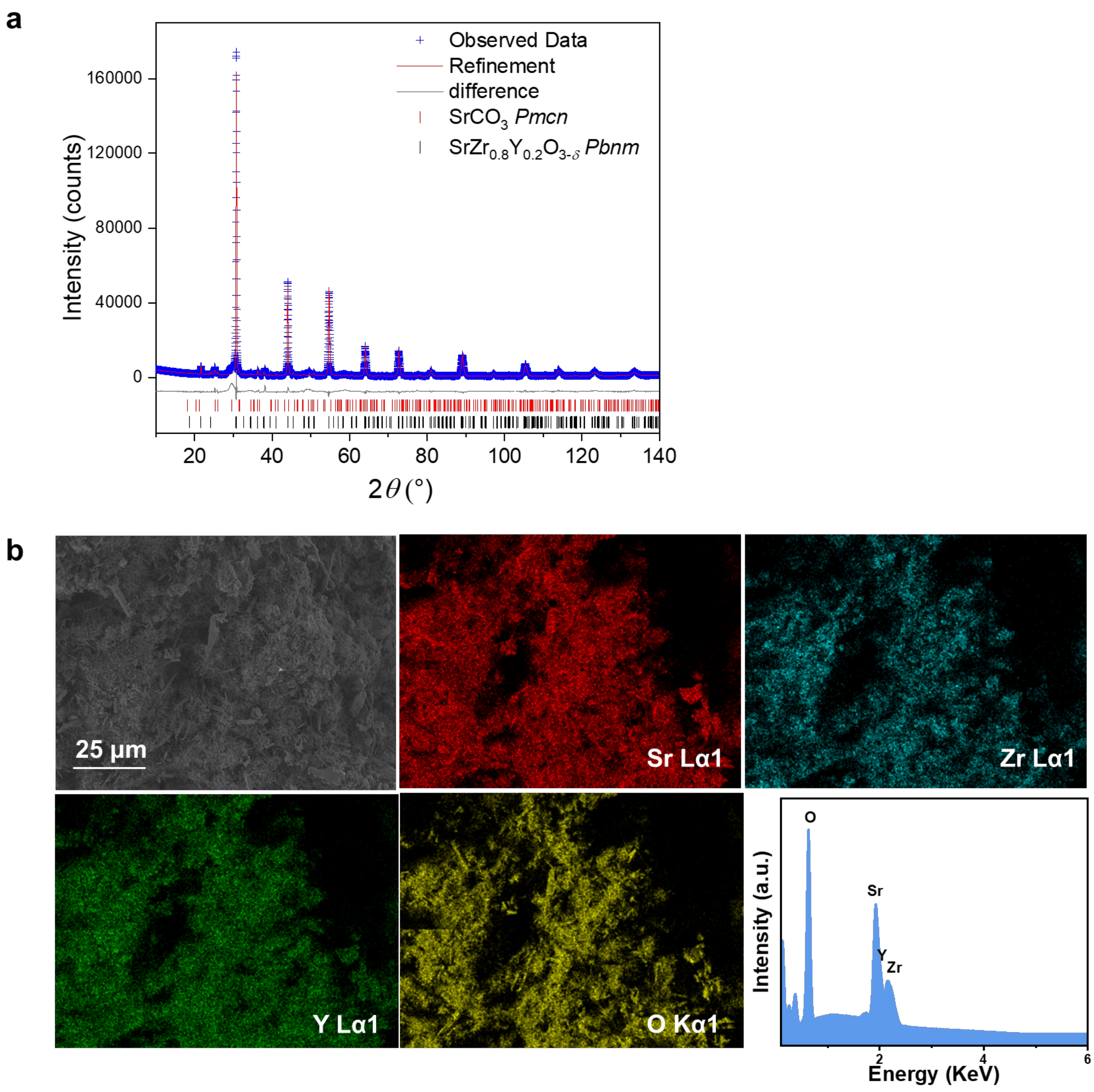

Supplementary Figure 15. Characterizations of SZYO20 in wet air. a, Experimental (crosses), calculated (solid red line), and difference (grey line) results of XRD refinement for SZYO20 after conductivity measurement in wet air. 98% $SrZr_{0.8}Y_{0.2}O_{3-\delta}$ and 2% $SrCO_3$. **b**, SEM image of SZYO20 pellet after the conductivity measurements in wet air at 25 μm resolution, corresponding EDX maps of Sr, Zr, Y, O elements and integrated EDX spectra from EDX element analysis.

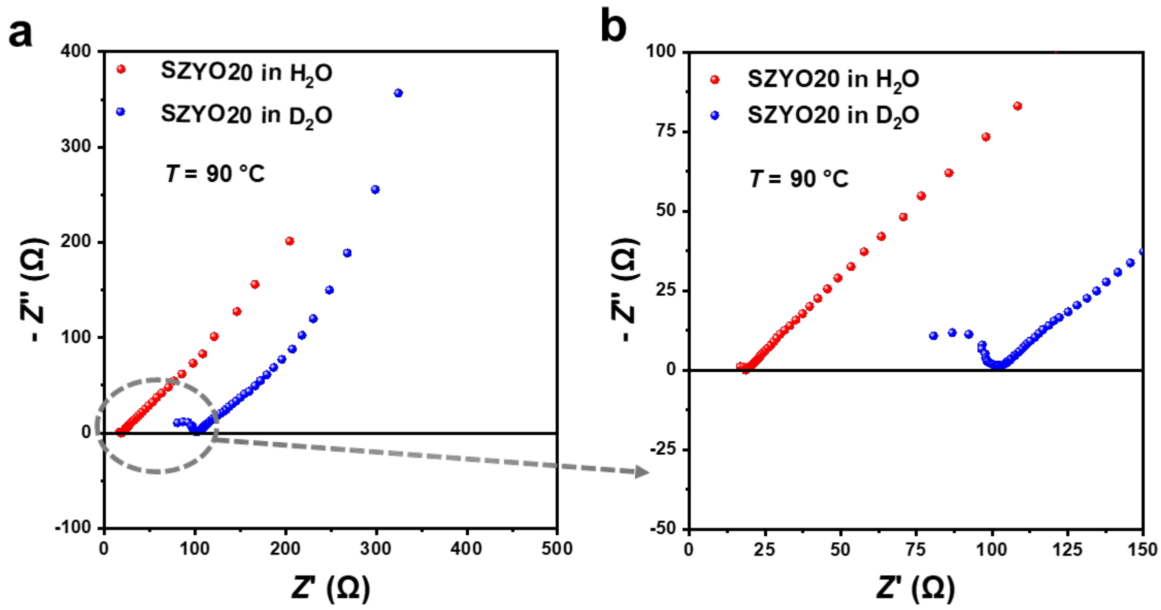

Supplementary Figure 16. Impedance spectra of SZYO20 sample. a, The representative a.c. impedance spectra of SZYO20 at 90 ℃ in H_2O and D_2O and **b**, Enlarged impedance spectra.

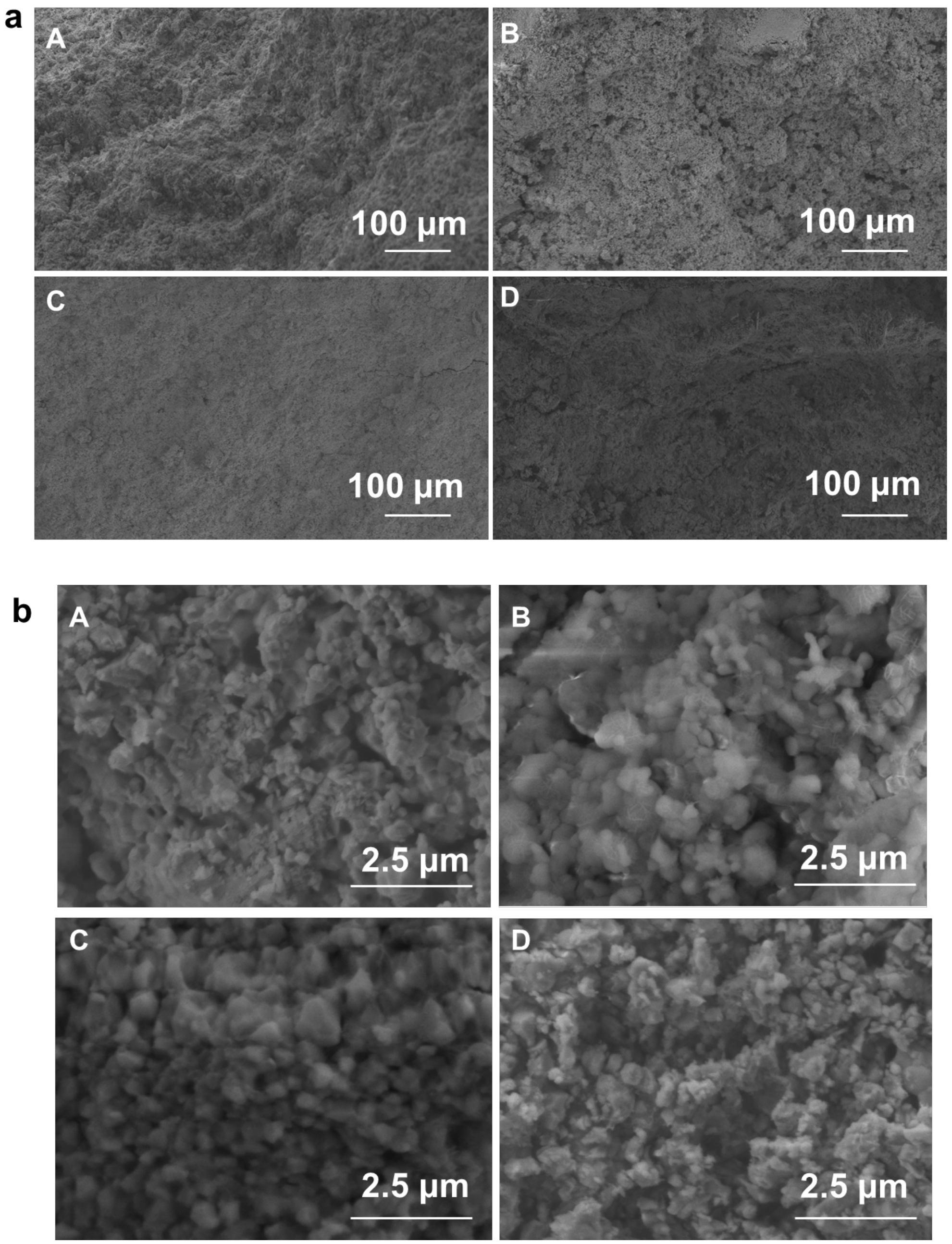

Supplementary Figure 17. SEM images of the cross section of SZYO20 pellets. The SEM image of (A) pure SZYO20 pellet, and after the conductivity measurements in (B) water, (C) D_2O and (D) wet air at **a**, 100 µm resolution, and **b**, 2.5 µm resolution, respectively.

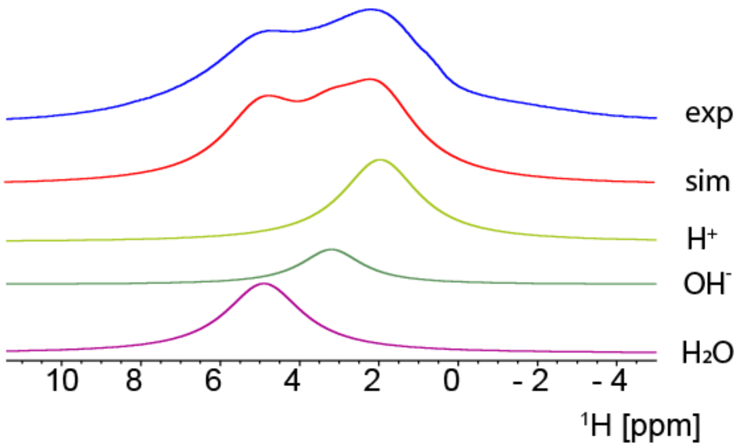

Supplementary Figure 18. Deconvolution of ^{1}H spectrum of the partially hydrated SZYO20.

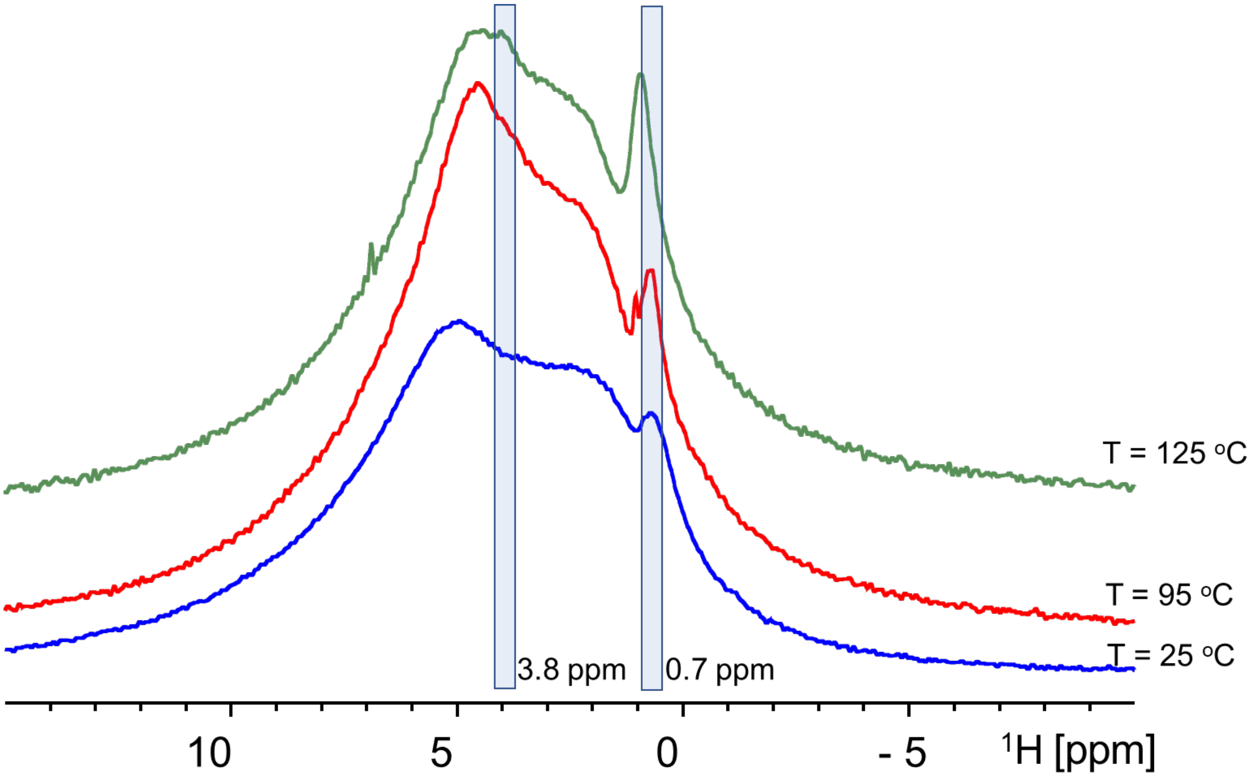

Supplementary Figure 19. ^{1}H MAS spectra at 25, 95 and 125 ^{o}C on partially hydrated SZYO20.

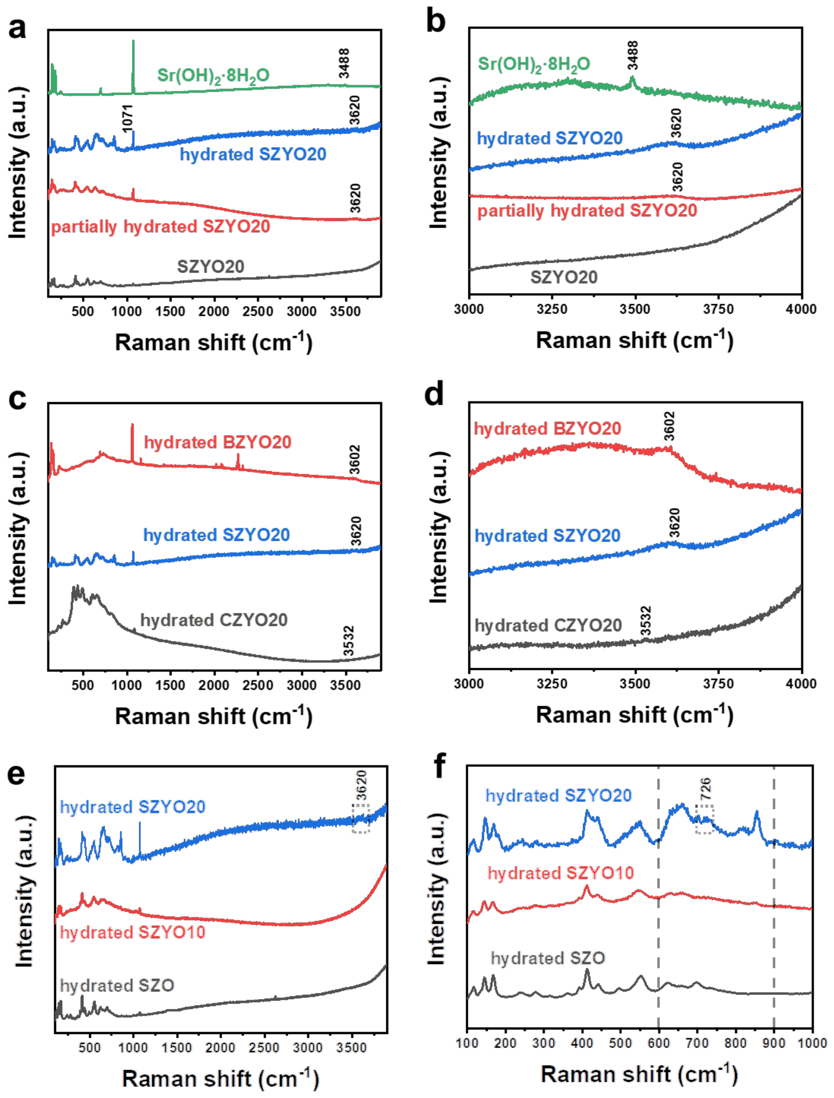

Supplementary Figure 20. Raman spectra of perovskite oxide pellets at room temperature. **a**,**b**, Raman spectra of SZYO20 samples and $Sr(OH)_2 \cdot 8H_2O$. (**a**) extended grating scan from 100 to 3900 cm^{-1}; (**b**) standard grating scan from 3000 to 4000 cm^{-1}. **c**,**d**, Raman spectra of $AZr_{0.8}Y_{0.2}O_{3-\delta}$ (A= Ca, Sr, Ba), (**c**) extended grating scan from 100 to 3900 cm^{-1}; (**d**) standard grating scan from 3000 to 4000 cm^{-1}. **e**,**f**, Raman spectra of $SrZr_{1-x}Y_xO_{3-\delta}$ (x=0, 0.1, 0.2), (**e**) extended grating scan from 100 to 3900 cm^{-1} and (**f**) enlarged spectra between 100 and 1000 cm^{-1}.

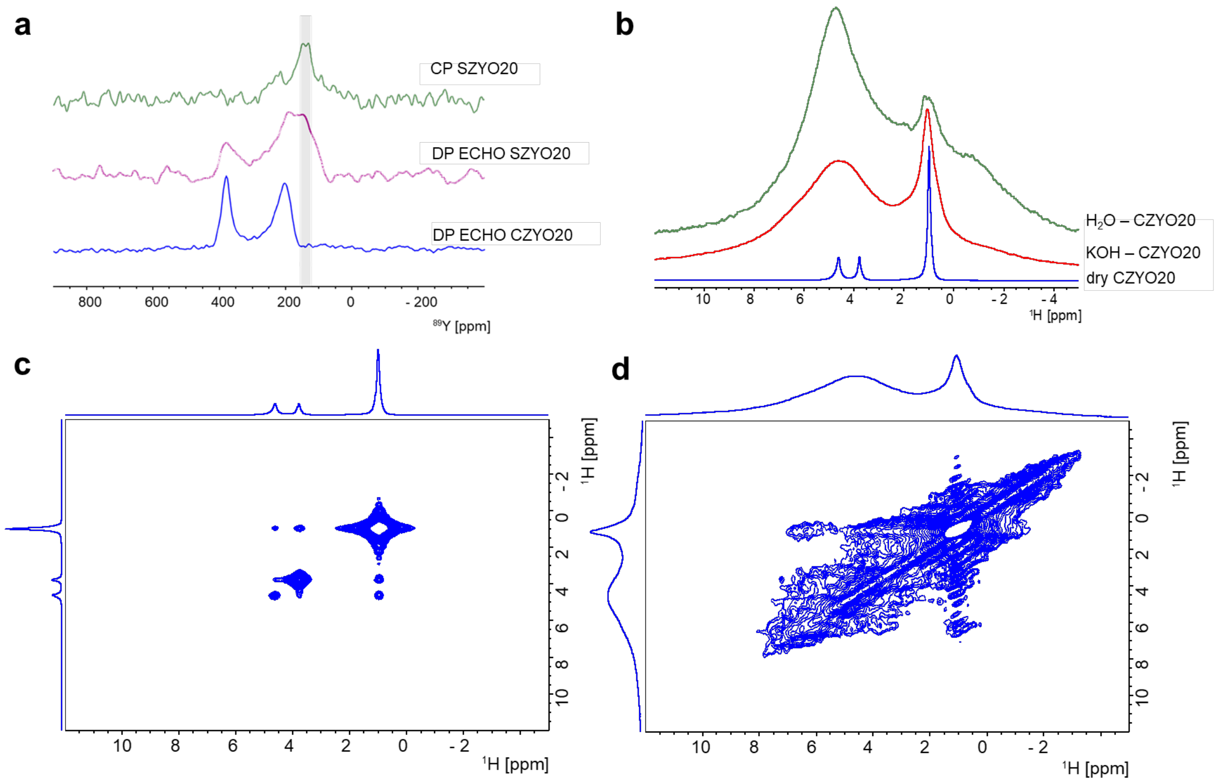

Supplementary Figure 21. Solid state NMR spectra of CZYO20. a, ^{89}Y DP spectrum (MAS at 8 kHz) measured with a spin echo of water-hydrated CZYO20 (blue), compared with the DP spectrum (purple) and ^{1}H-^{89}Y cross polarization (green) spectrum of partially hydrated SZYO20. **b**, The solid state ^{1}H NMR spectra of dry (blue), water-hydrated CZYO20 (green) and KOH-hydrated (red) CZYO20. **c**,**d**, ^{1}H-^{1}H NOESY correlation spectra (MAS 60 kHz) of dry (**c**) and KOH-hydrated (**d**) CZYO20.

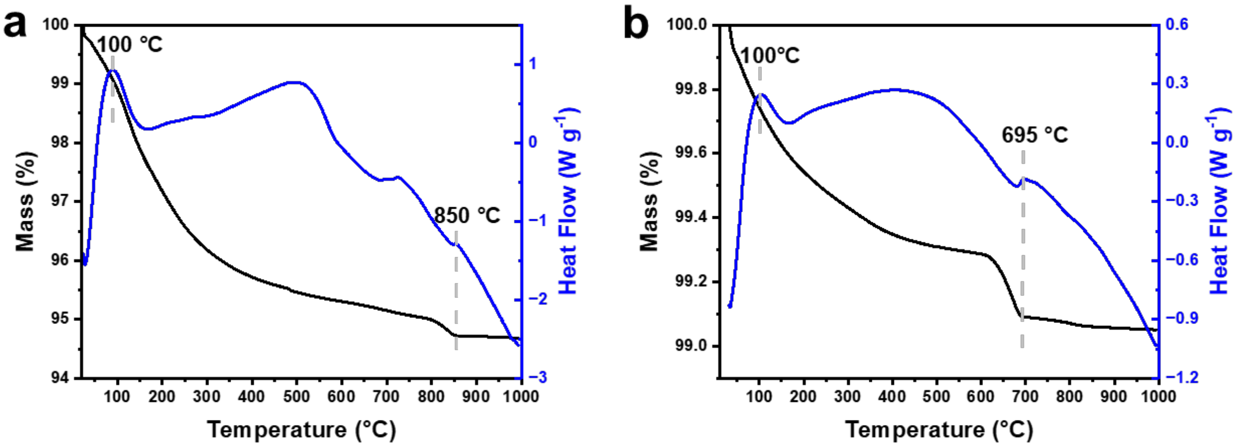

Supplementary Figure 22. The TG/DSC curves of hydrated perovskite oxide powders. a, Partially hydrated SZYO20. **b**, Water-hydrated CZYO20**.**

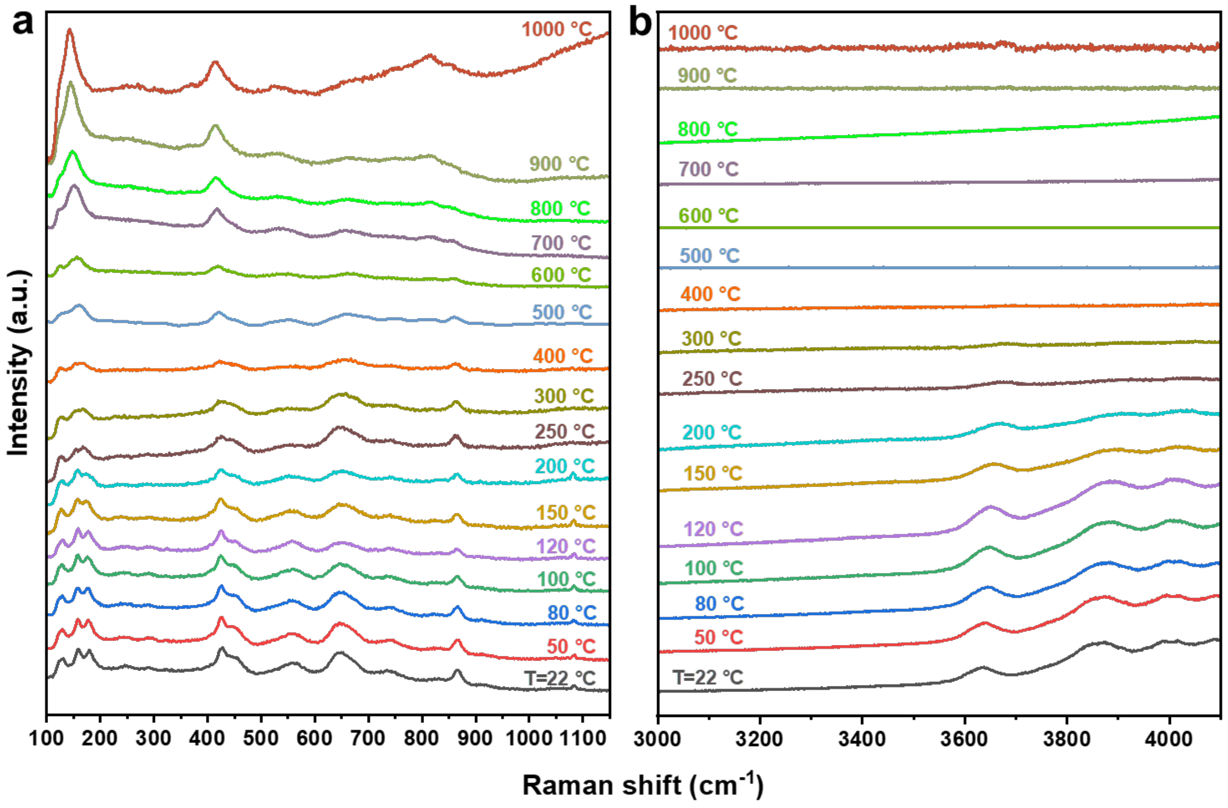

Supplementary Figure 23. Variable temperature Raman spectra measured in situ for partially hydrated SZYO20 powders. a, In the lattice frequency region. **b**, In the O-H stretch frequency region.

5

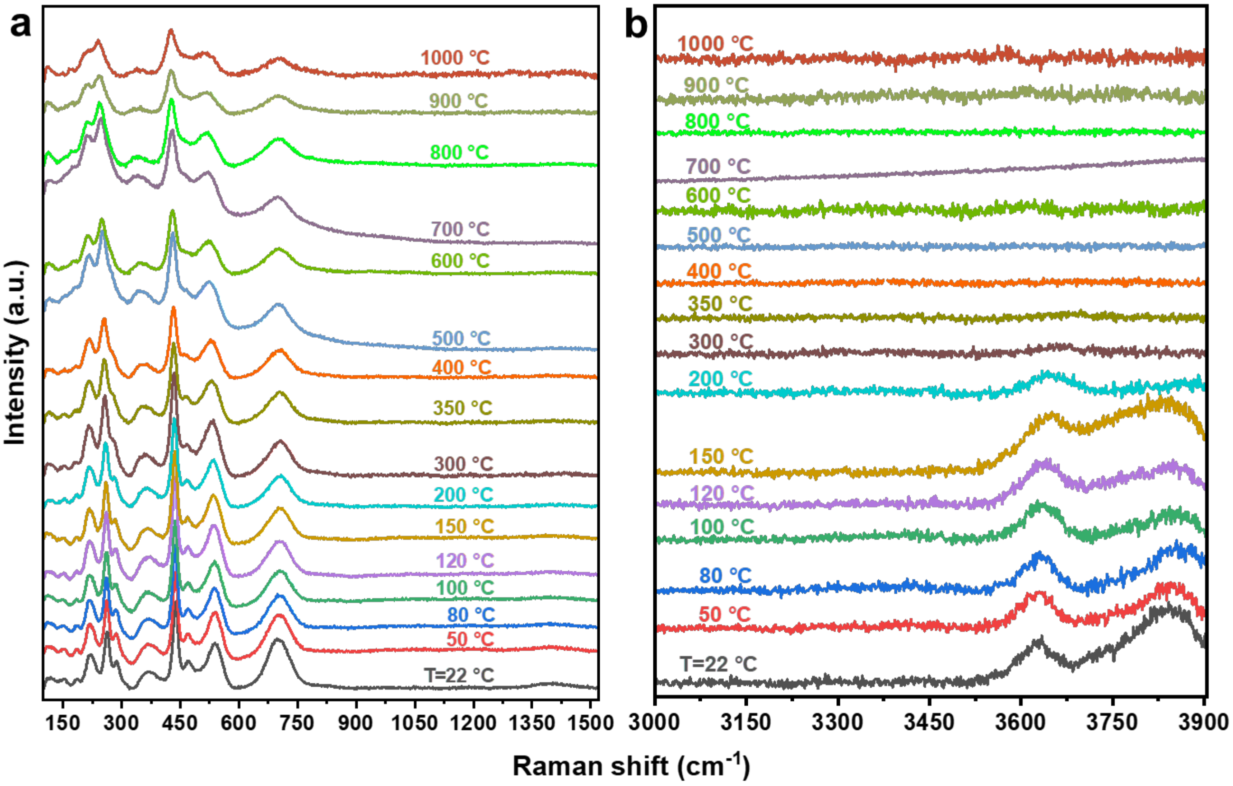

Supplementary Figure 24. Variable temperature Raman spectra measured in situ for water-hydrated CZYO20 powders. **a**, In the lattice frequency region. **b**, In the O-H stretch frequency region.

5

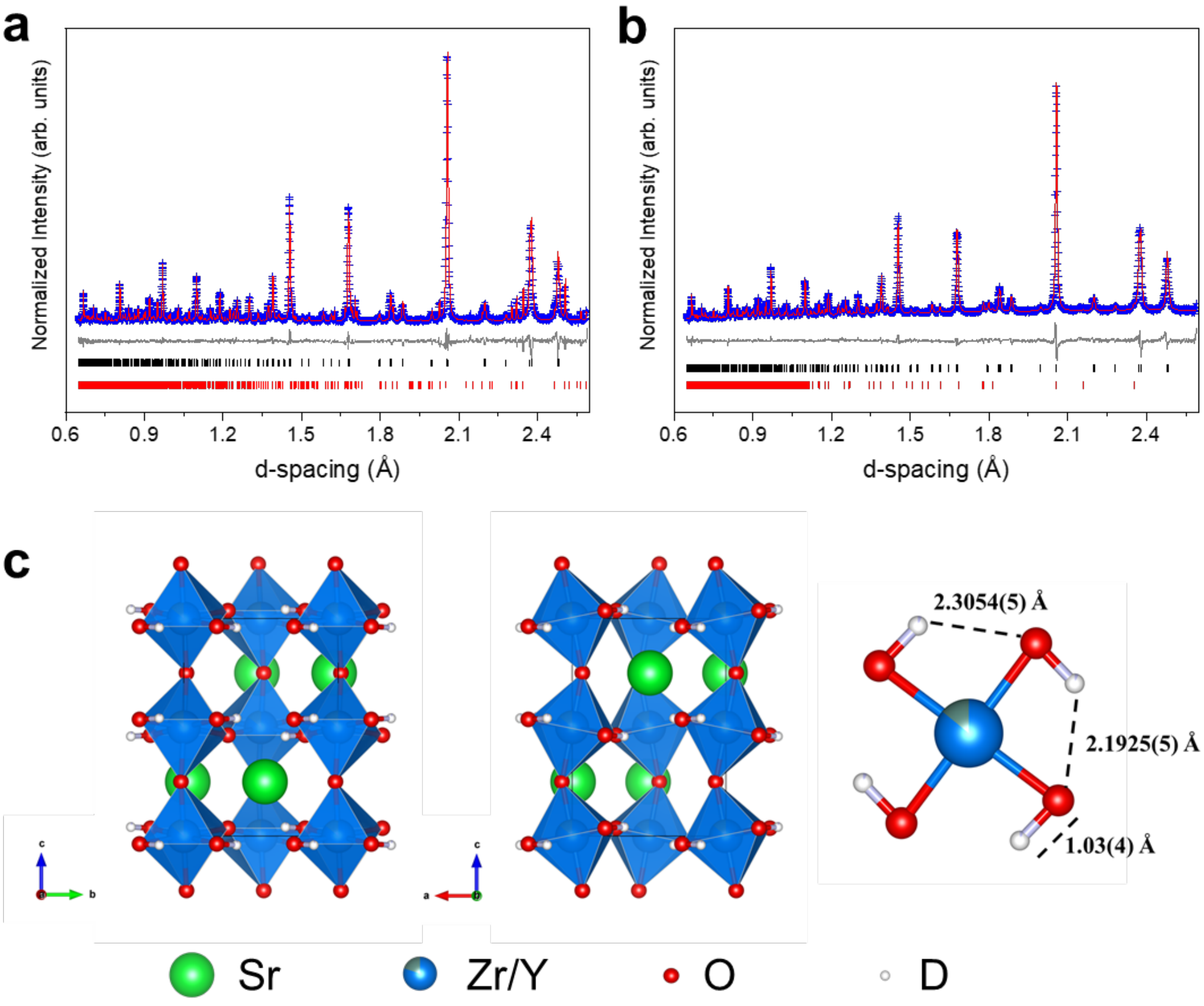

Supplementary Figure 25. Neutron diffraction patterns for SZYO20 powders. a, Rietveld fit of vacuum dried SZYO20 (black tick marks) against Bank 1 NPD data collected on HRPD. Red tick marks are from the SrY_2O_4 (9.61%) secondary phase. **b**, Rietveld fit of deuterated SZYO20 (black tick marks) against Bank 1 NPD data collected on HRPD. Red tick marks are from a $Y(OH(D))_3$ (6.9%) secondary phase. **c**, The structure of $SrZr_{0.86}Y_{0.14}O_{2.86}(OD)_{0.14}$ refined from NPD data, shown viewed down the (001), (010) axes and illustration of the O-D distances.

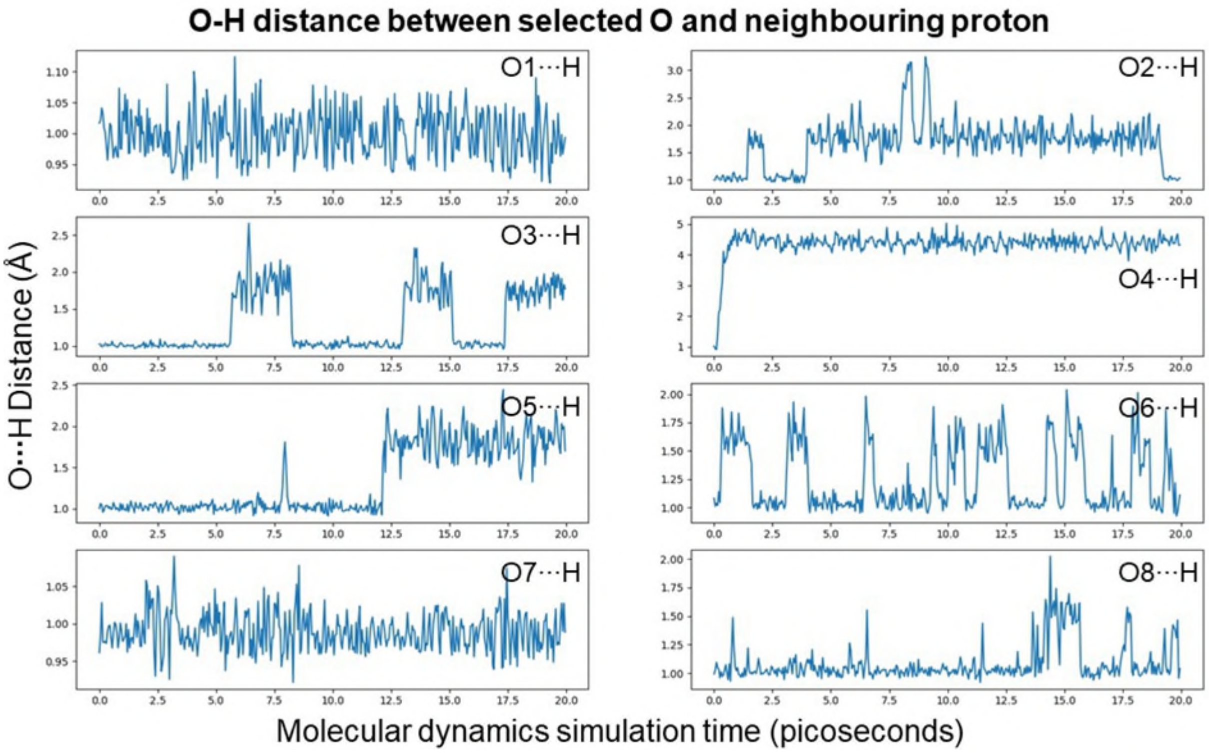

Supplementary Figure 26. Distances (in Å) between the 8 protons and their neighbouring O atoms as a function of MD simulation time (in ps).

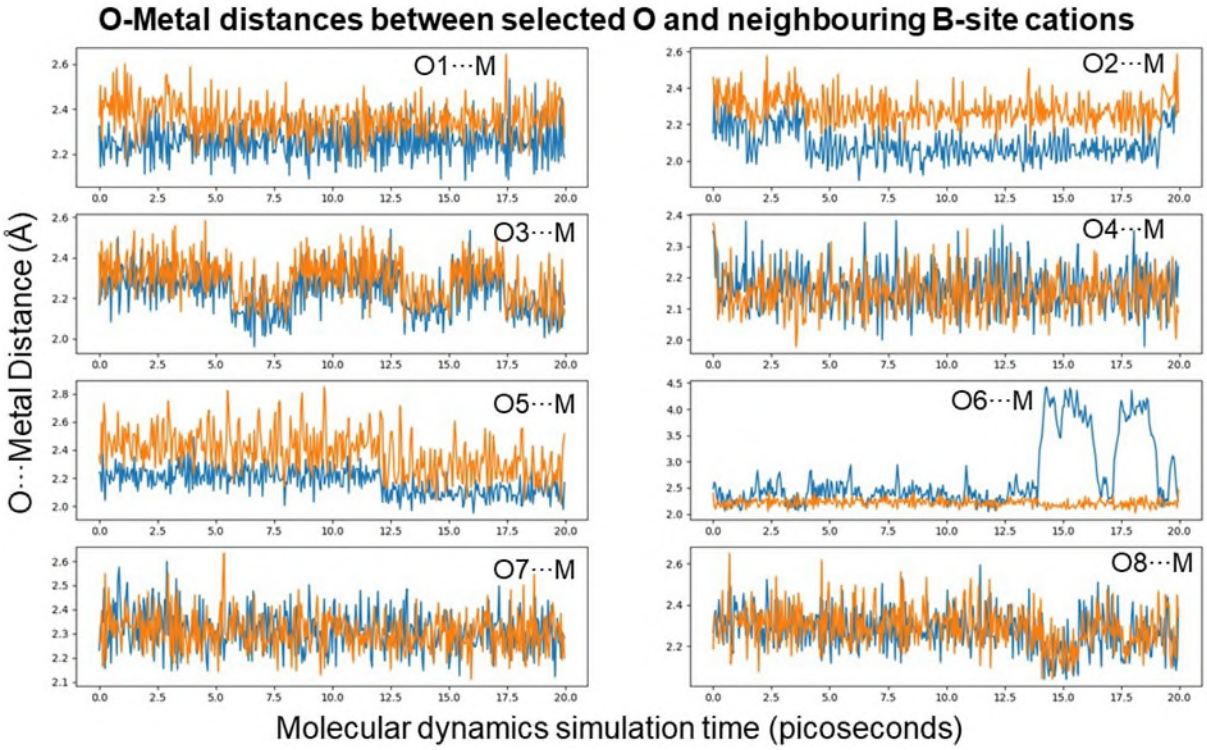

Supplementary Figure 27. Distances (in Å) between the 8 oxygen atoms that were bonded to protons initially, and the neighbouring B-site cations as a function of MD simulation time (in ps). The blue and orange lines represent the distances between an O^{2-} anion and its two neighbouring B-site cations, respectively.

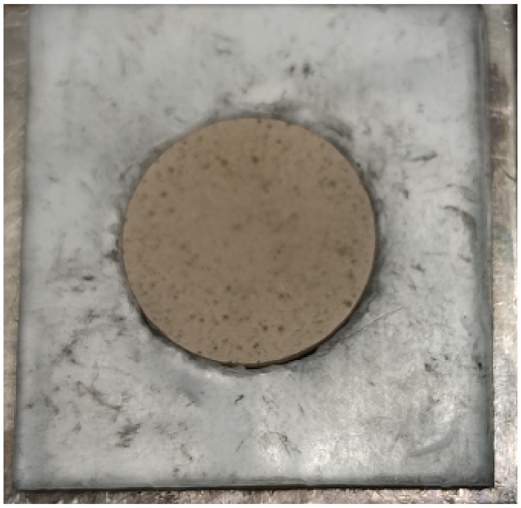

Supplementary Figure 28. Photo of a SZYO20 electrolyte. The electrolyte pellet was sealed by gaskets to be assembled in the fuel cell jig.

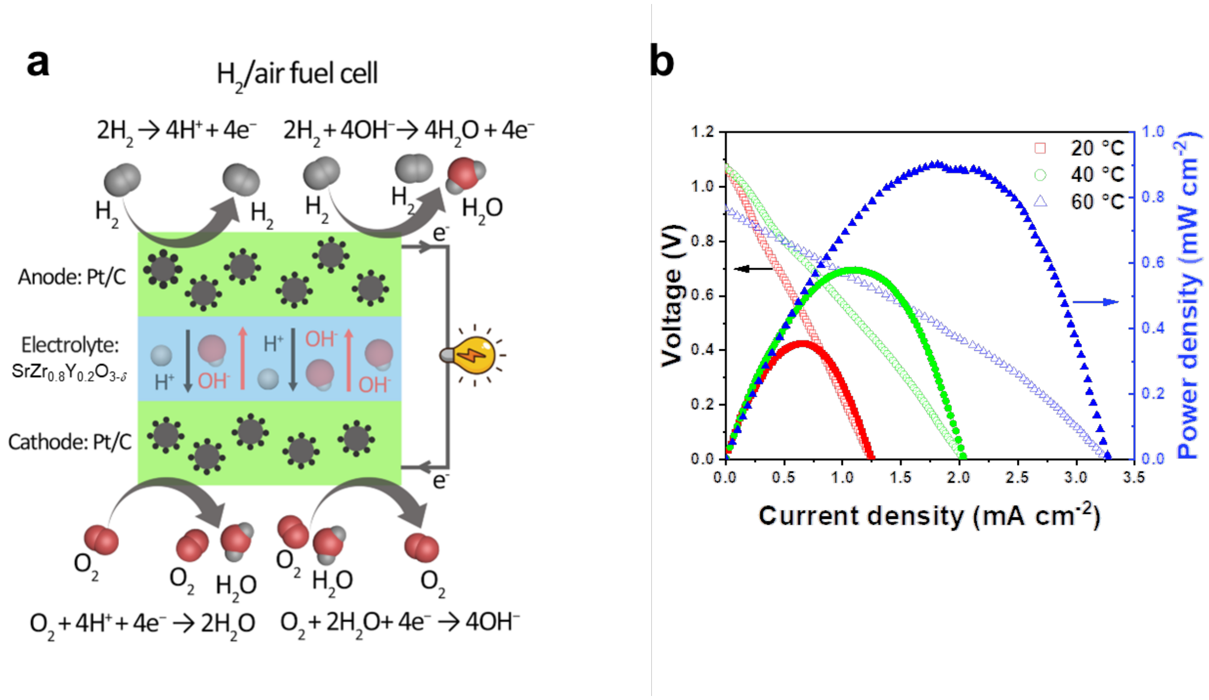

Supplementary Figure 29. Fuel cell demonstration when SZYO20 was used as electrolyte. **a**, Working principle of low temperature SOFCs for a H_2/air fuel cell. **b**, The performance of a H_2/air fuel cells at different temperatures.

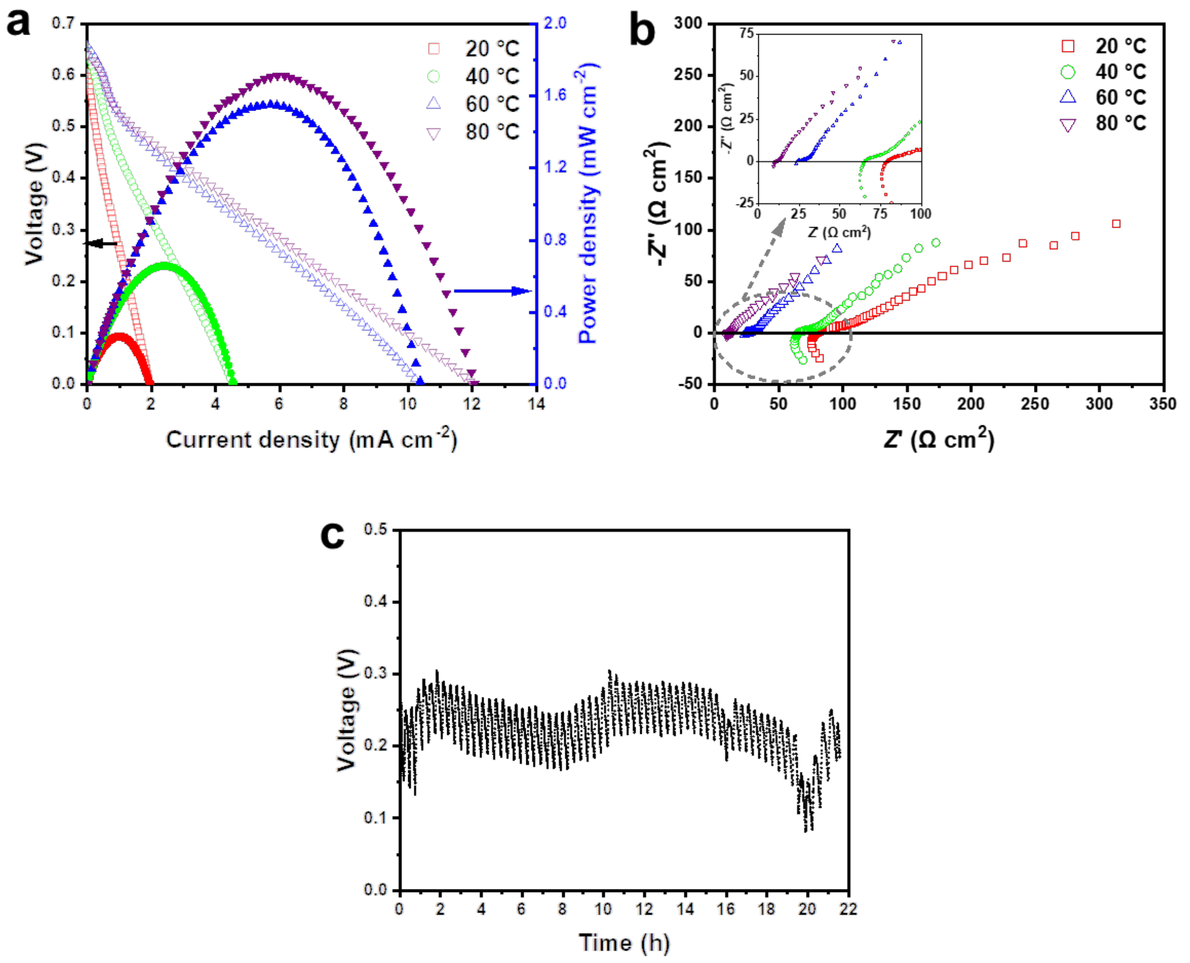

Supplementary Figure 30. Fuel cell demonstration when SZYO20 was used as electrolyte. **a**,**b**, (**a**) Fuel cell performance and (**b**) a.c. impedance of an NH_3/air fuel cell using SZYO20 pellet as electrolyte. Enlarged impedance spectra is displayed in insert. 35% NH_3H_2O solution was used as a fuel. PtIr as anode and Pt as cathode. **c**, The durability test of the NH_3/air fuel cell using SZYO20 electrolyte. Test conditions: at 4 mA cm^{-2}, cell temperature of 60 °C.

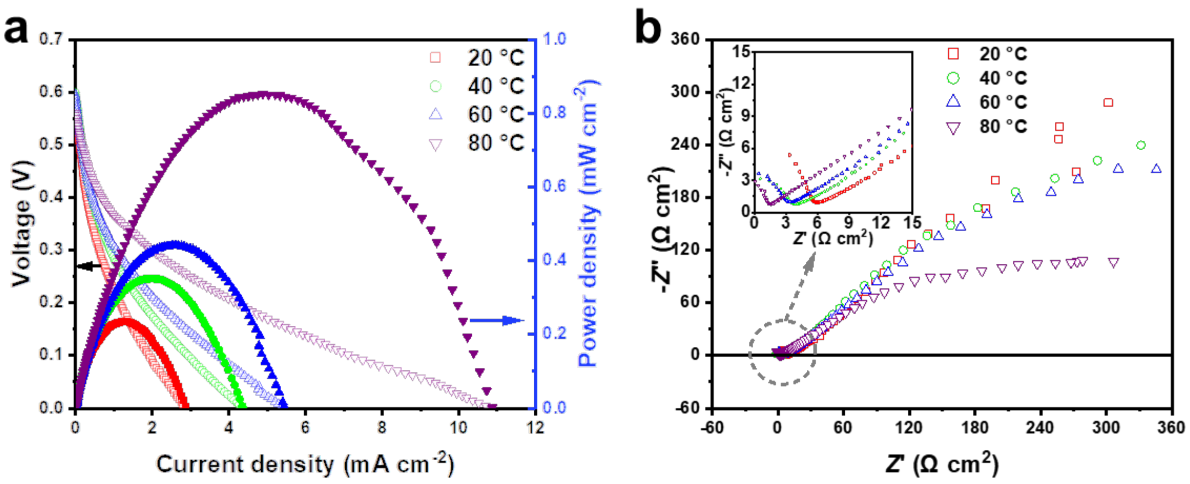

Supplementary Figure 31. Fuel cell demonstration when AEM were used as electrolyte. a,**b**, (**a**) Fuel cell performance and (**b**) a.c. impedance of an NH_3/air fuel cell using SZYO20 pellet as electrolyte. Enlarged impedance spectra is displayed in insert. 35% NH_3H_2O solution was used as a fuel. PtIr as anode and Pt as cathode.

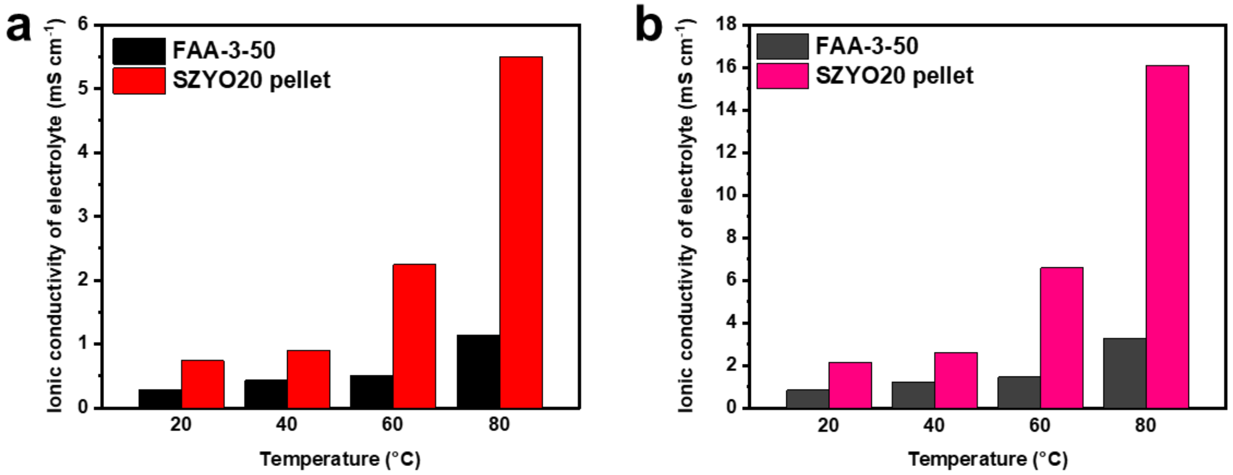

Supplementary Figure 32. Ionic conductivity of SZYO20 and AEM electrolyte in the direct ammonia fuel cell (35 wt% NH_3H_2O solution as the fuel). a, Calculation according to total area of the electrolyte pellet including the parts without electrode. **b**, Calculation according to the cell area coated with electrode only, of 1 cm^2.

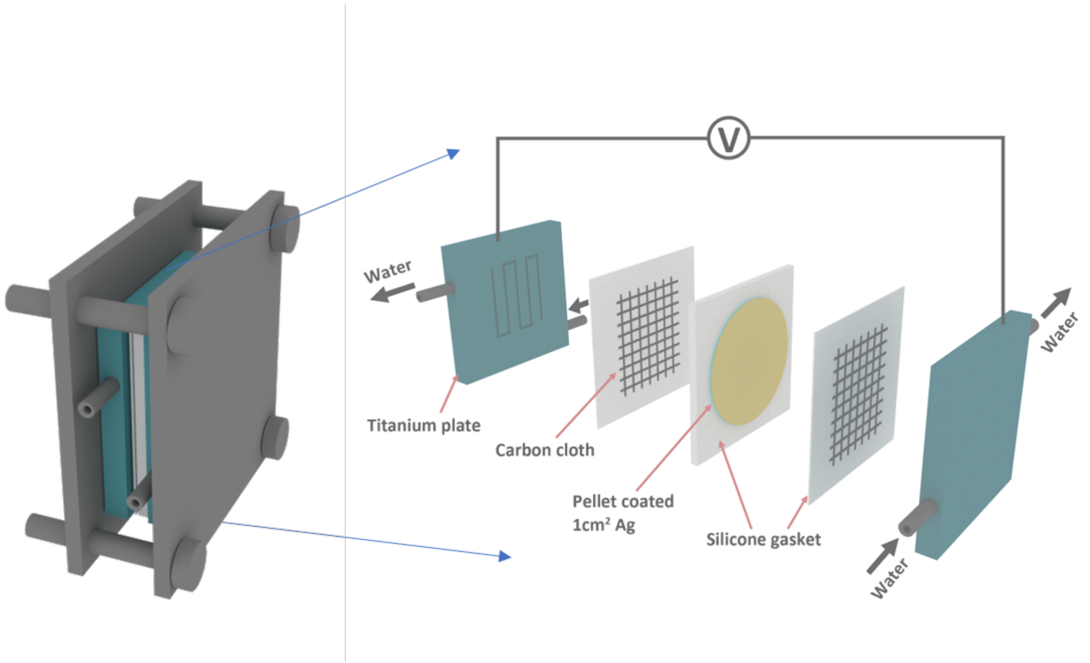

Supplementary Figure 33. Schematic diagram of the home-made fuel cell set-up to be used in fuel cell measurement, conductivity measurement and zero-gap electrolyser.

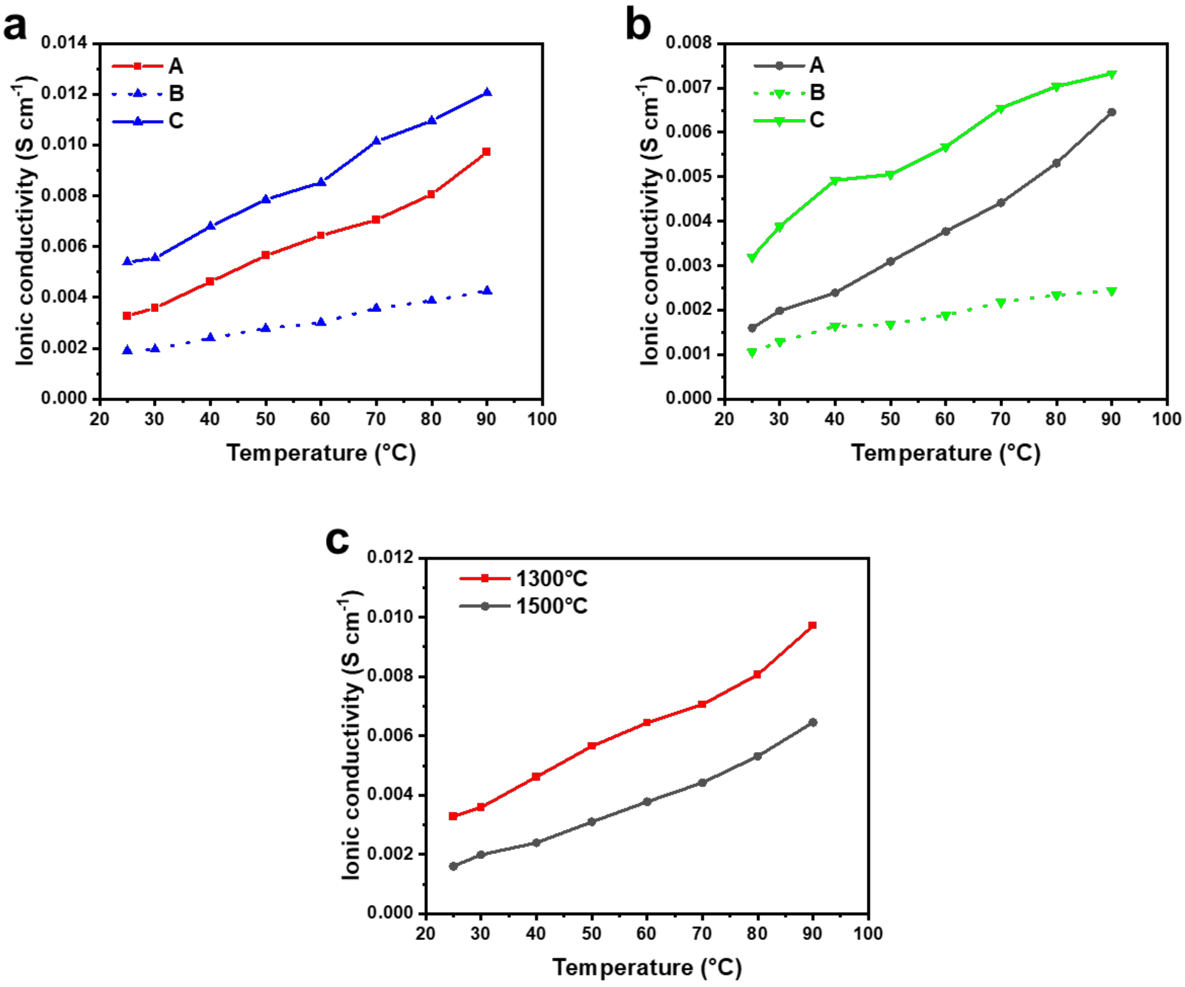

Supplementary Figure 34. The ionic conductivity of SZYO20 pellets in water. a, Pellets were sintered at 1300 °C. **b**, Pellets were sintered at 1500 °C. In figures, (A) Conductivity was measured in the set-up shown in Supplementary Fig. 5; (B) Conductivity was measured in the set-up shown in Supplementary Fig. 33, the conductive area is accounted from the cross-sectional area of pellet; (C) Conductivity was measured in the set-up shown in Supplementary Fig. 33, the conductive area is accounted from the active area of silver paste on pellet, i.e., 1 cm^{-2}. **c**, Comparison of ionic conductivity in water of SZYO20 sintered at different temperatures.

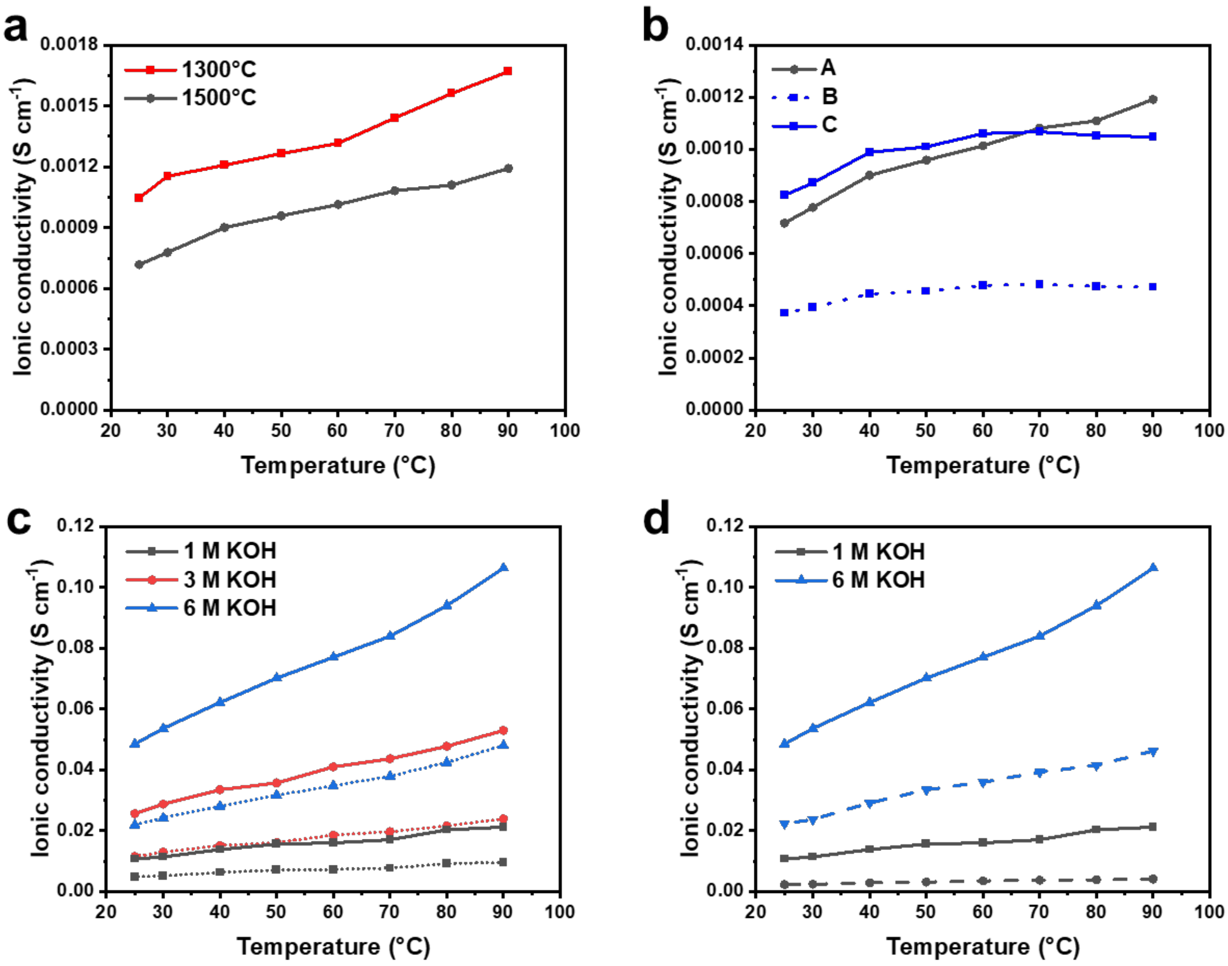

Supplementary Figure 35. The ionic conductivity of CZYO20 pellets. a, Comparison of ionic conductivity in water of CZYO20 sintered at different temperatures. **b**, Ionic conductivity of CZYO20 sintered at 1500 °C in water. In the figure, (A) Conductivity was measured in the set-up shown in Supplementary Fig. 5; (B) Conductivity was measured in the set-up shown in Supplementary Fig. 33, the conductive area is accounted from the cross-sectional area of pellet; (C) Conductivity was measured in the set-up shown in Supplementary Fig. 33, the conductive area is accounted from the active area of silver paste on pellet, i.e., 1 cm^{-2}. **c**, Conductivity of CZYO20 sintered at 1500 °C, measured in the set-up shown in Supplementary Fig. 33, using

different concentration of KOH passing through the set-up. The conductive area is accounted from the cross-sectional area of pellet (short dot line) or the active area of silver paste on pellet, i.e., 1 cm^{-2} (solid line). **d**, Comparison of ionic conductivity of CZO (dash line) and CZYO20 (solid line) sintered at 1500 °C in KOH solutions, measured in the set-up shown in Supplementary Fig. 33. The conductive area is accounted from the active area of silver paste on pellet, i.e., 1 cm^{-2}.

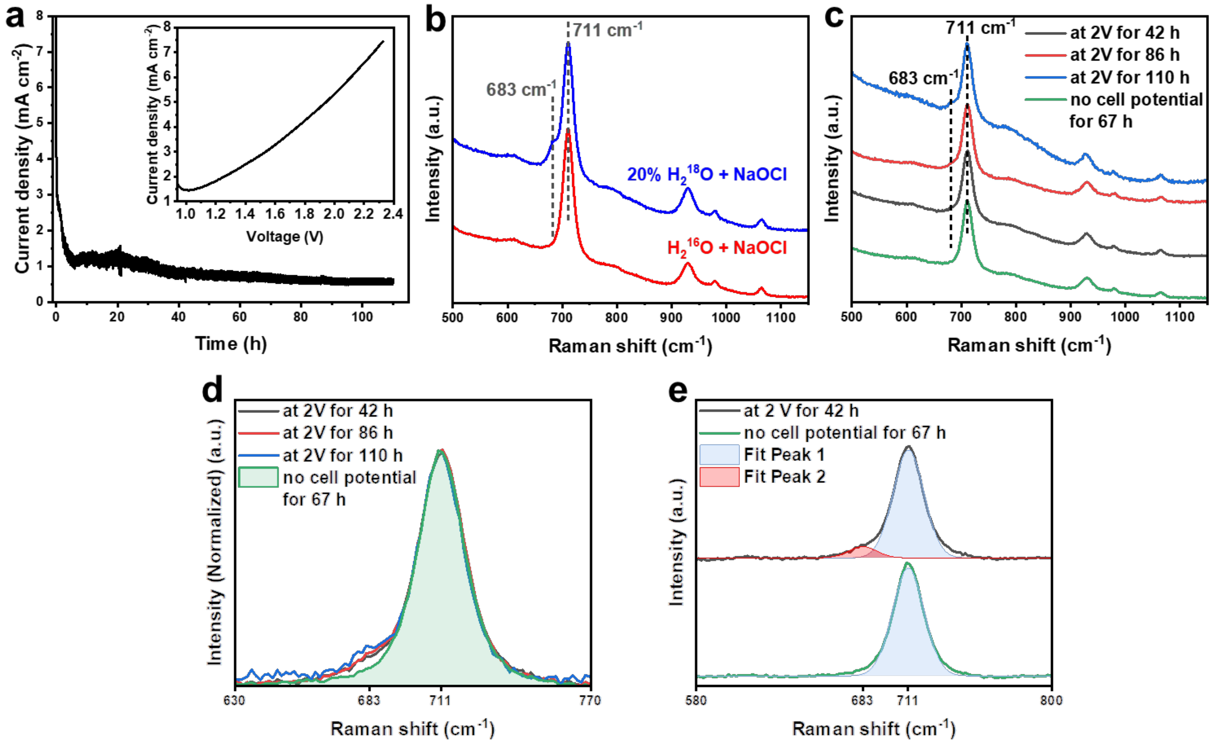

Supplementary Figure 36. Water electrolyser. HER-20% $H_2{}^{18}O$ in $H_2{}^{16}O$, OER-$H_2{}^{16}O$. a, The durability test of the water electrolyser using dense CZYO20 electrolyte. Test conditions: at 2 V, cell temperature of 90 °C. Polarization curve is displayed insert. **b**, Raman spectra of NaOCl diluted in 20% $H_2{}^{18}O$ and in $H_2{}^{16}O$. **c-e**, Raman spectra of solution in OER side of electrolyser, the samples were taken from different electrolysis durations, NaOCl was added in samples before Raman measurement (1 mL NaOCl solution + 3 mL sample in OER side), (**c**) standard grating scan from 500 to 1150 cm^{-1}; (**d**) enlarged normalized spectra to present the difference in the band at 683 cm^{-1}; (**e**) enlarged spectra with the corresponding curve fitted bands. The *x* centre of Fit Peak 1 is at 711 cm^{-1}, the *x* centre of Fit Peak 2 is at 683 cm^{-1}, the R-Square (COD) for fitting curves is 0.994 for black line and 0.993 for green line respectively.

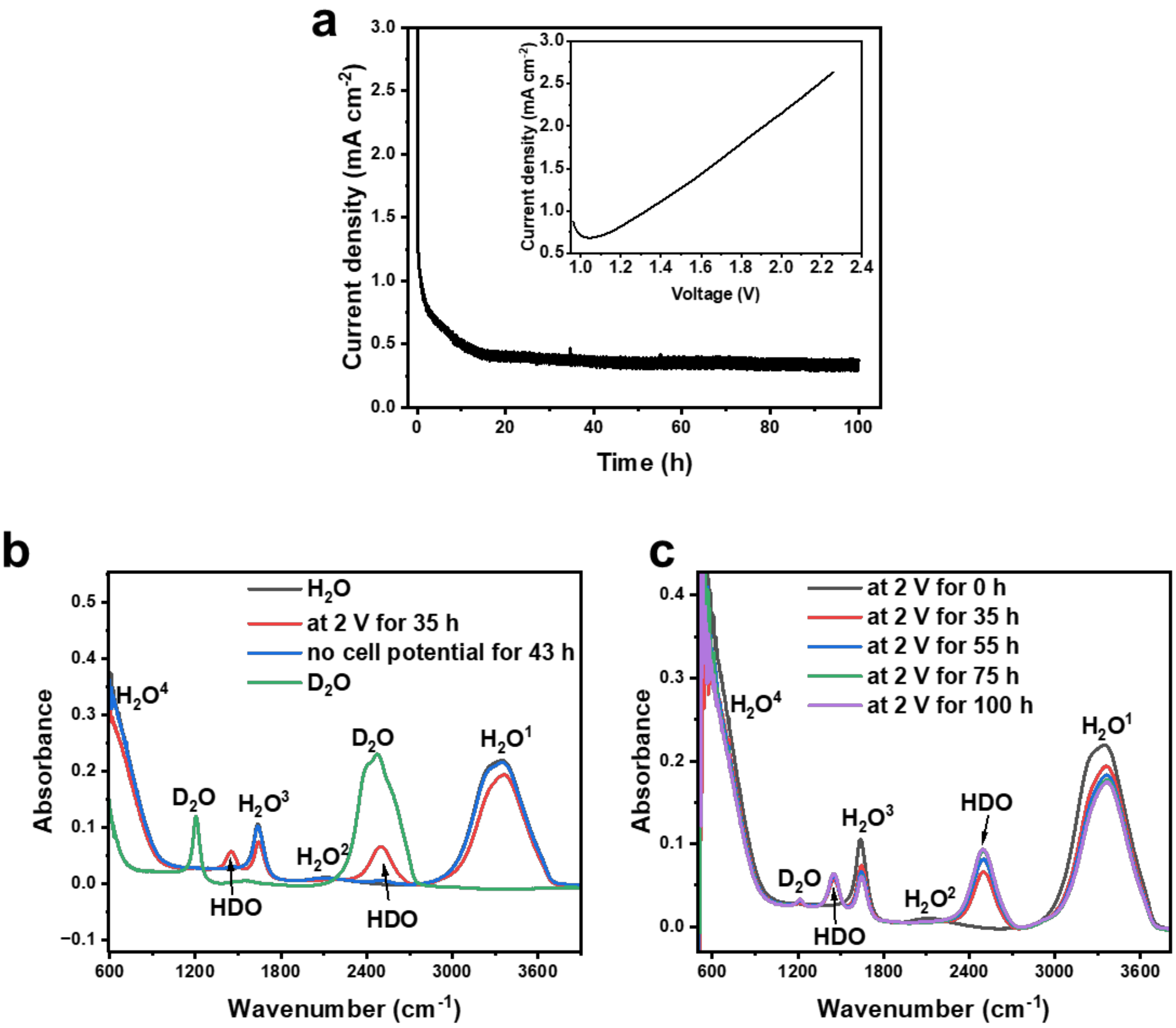

Supplementary Figure 37. Water electrolyser. HER-D_2O, OER-H_2O. a, The durability test of the water electrolyser using dense CZYO20 electrolyte. Test conditions: at 2 V, cell temperature of 90 °C. Polarization curve is displayed insert. **b**, ATR-FTIR spectra of solution in OER side of electrolyser, compared with the spectra of pure H_2O and D_2O. **c**, ATR-FTIR spectra of solution in OER side of electrolyser, the samples were taken from different electrolysis durations at 2 V (the four H_2O absorbances denoted 1-4).

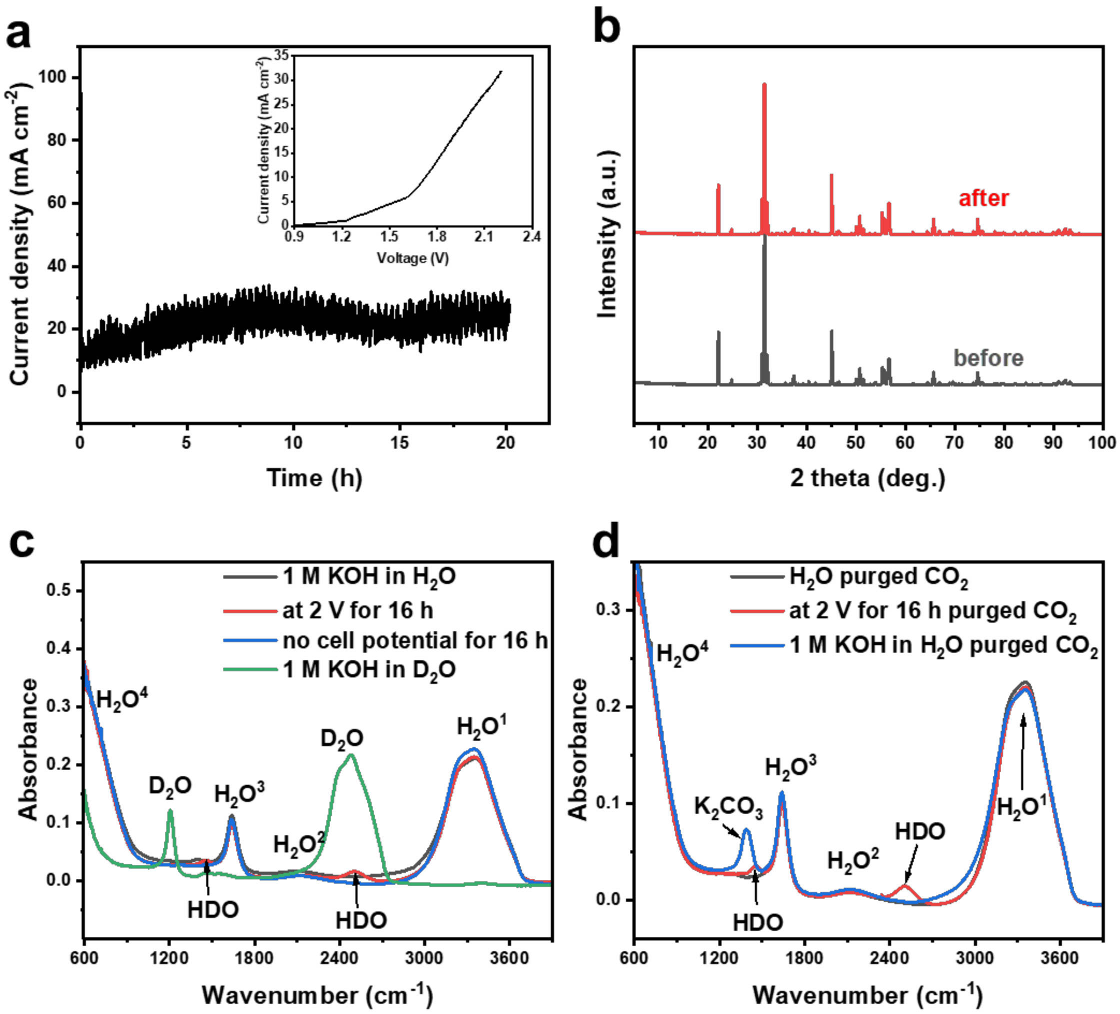

Supplementary Figure 38. Water electrolyser. HER-1 M KOH in D_2O, OER-H_2O. a, The durability test of the water electrolyser using dense CZYO20 electrolyte. Test conditions: at 2 V, cell temperature of 80 °C. Polarization curve is displayed insert. **b**, XRD pattern of CZYO20 electrolyte before and after water electrolysis. **c**, ATR-FTIR spectra of solution in OER side of electrolyser, compared with the spectra of 1 M KOH in pure H_2O and D_2O. **d**, ATR-FTIR spectra of solution in OER side of electrolyser, purged CO_2 to detect KOH crossover.

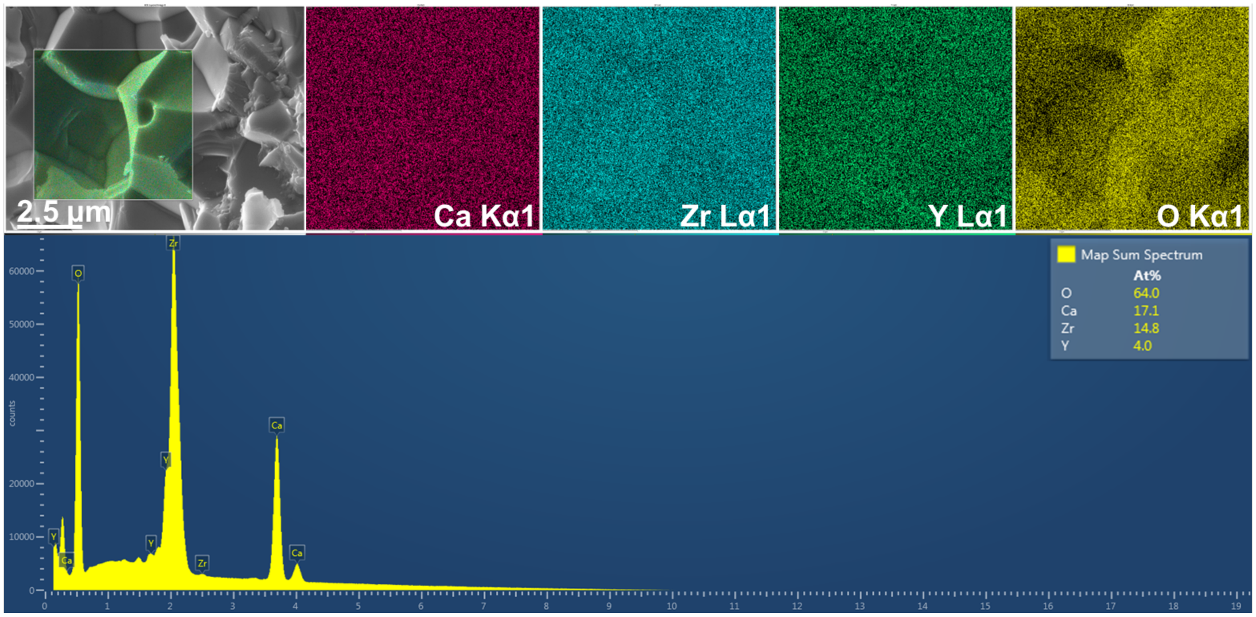

Supplementary Figure 39. SEM image of CZYO20 pellet sintered at 1500 °C at 2.5 µm resolution, corresponding EDX maps of Ca, Zr, Y, O elements and integrated EDX spectra from EDX element analysis.

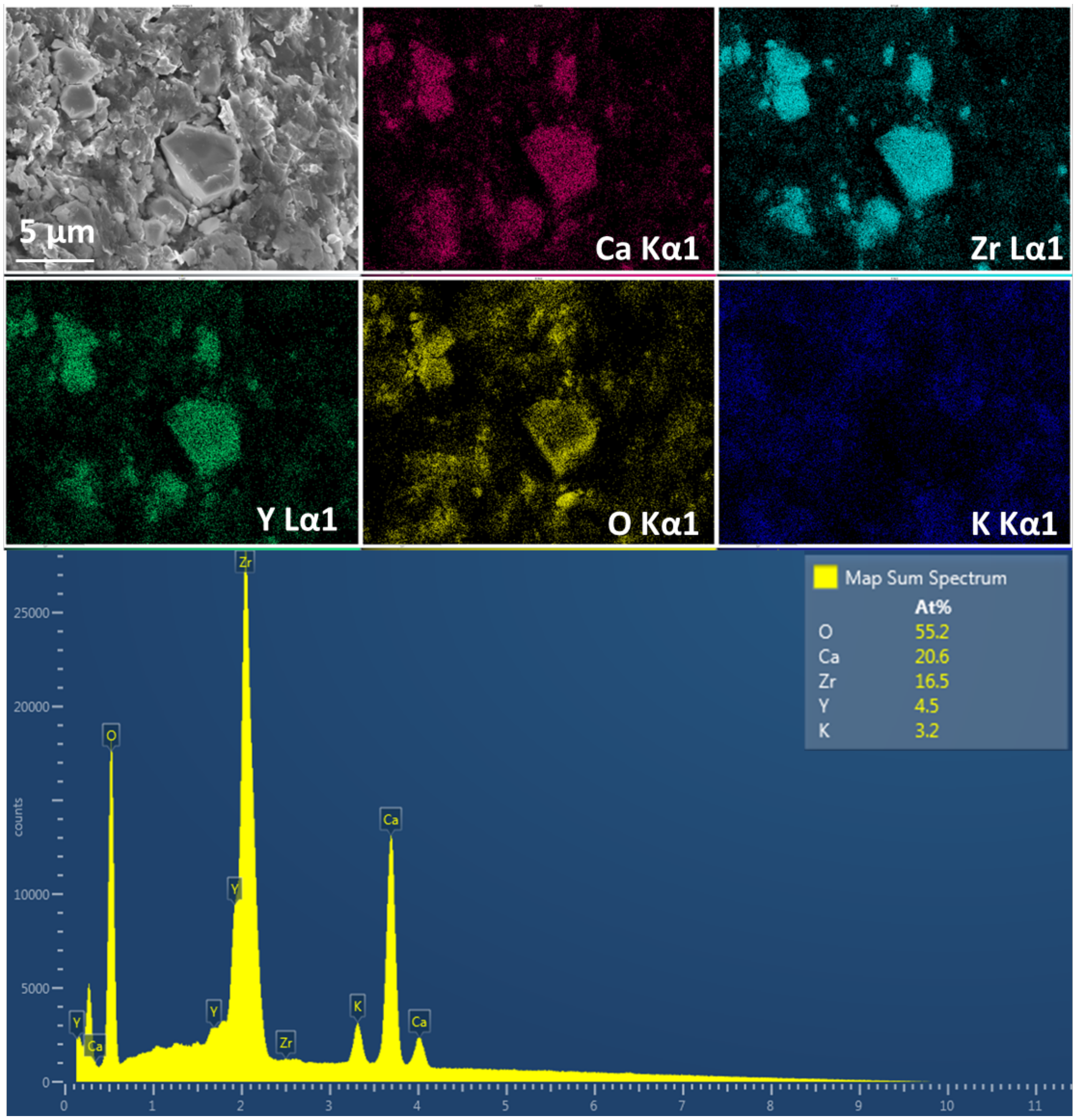

Supplementary Figure 40. SEM image of dense CZYO20 pellet after the measurements in KOH at 5 µm resolution, corresponding EDX maps of Ca, Zr, Y, O, K elements and integrated EDX spectra from EDX element analysis.

Supplementary Tables 1-9

Supplementary Table 1. Crystallographic refinement parameters of perovskite oxides.

Crystallographic parameters	$SrZrO_3$	$SrZr_{0.9}Y_{0.1}O_{3-\delta}$	$SrZr_{0.8}Y_{0.2}O_{3-\delta}$	$SrZr_{0.8}Y_{0.2}O_{3-\delta}$ after treated in H_2O	$SrZr_{0.8}Y_{0.2}O_{3-\delta}$ after conductivity test in wet air	$CaZr_{0.8}Y_{0.2}O_{3-\delta}$	$BaZr_{0.8}Y_{0.2}O_{3-\delta}$
Crystal system	Orthorhombic	Orthorhombic	Orthorhombic	Orthorhombic	Orthorhombic	Orthorhombic	Cubic
Space group	*Pbnm* (62)	*Pbnm* (62)	*Pbnm* (62)	*Pbnm* (62)	*Pbnm* (62)	*Pcmn* (62)	*Pm-3m* (221)
a (Å)	5.79665(5)	5.80406(16)	5.8132(5)	5.8104(4)	5.8023(3)	5.59859(7)	4.21008(11)
b (Å)	5.81625(5)	5.82832(22)	5.8305(4)	5.8304(3)	5.8267(3)	8.04465(10)	4.21008(11)
c (Å)	8.20461(8)	8.21380(22)	8.2231(7)	8.2279(6)	8.2179(6)	5.77915(7)	4.21008(11)
V (Å^3)	276.617(4)	277.855(16)	278.711(21)	278.739(16)	277.832(18)	260.286(6)	74.623(6)
Position							
Sr	*x* = -0.0041(3);<br>*y* = 0.52326(9);<br>*z* = 0.25	*x* = 0.0003(6)<br>*y* = 0.5266(3);<br>*z* = 0.25	*x* = -0.0043(12)<br>*y* = 0.5210(5)<br>*z* = 0.25	*x* = 0.0148(13);<br>*y* = 0.5244(3);<br>*z* = 0.25	*x* = -0.0084(9);<br>*y* = 0.5238(4);<br>*z* = 0.25	Ca: *x* = 0.0145(5);<br>*y* = 0.25;<br>*z* = 0.04991(29)	Ba: *x* = 0.5;<br>*y* = 0.5;<br>*z* = 0.5
Zr	*x* = 0;<br>*y* = 0;<br>*z* = 0	*x* = 0;<br>*y* = 0;<br>*z* = 0	*x* = 0;<br>*y* = 0;<br>*z* = 0	*x* = 0;<br>*y* = 0;<br>*z* = 0	*x* = 0;<br>*y* = 0;<br>*z* = 0	*x* = 0;<br>*y* = 0;<br>*z* = 0.5	*x* = 0;<br>*y* = 0;<br>*z* = 0
Y	/	*x* = 0;<br>*y* = 0;<br>*z* = 0	*x* = 0;<br>*y* = 0;<br>*z* = 0	*x* = 0;<br>*y* = 0;<br>*z* = 0	*x* = 0;<br>*y* = 0;<br>*z* = 0	*x* = 0;<br>*y* = 0;<br>*z* = 0.5	*x* = 0;<br>*y* = 0;<br>*z* = 0
O1	*x* = 0.0589(11);<br>*y* = -0.0131(6);<br>*z* = 0.25	*x* = 0.0467(11);<br>*y* = 0.9964(9);<br>*z* = 0.25	*x* = 0.06474;<br>*y* = -0.01495;<br>*z* = 0.25	*x* = -0.017711;<br>*y* = 0.035282;<br>*z* = 0.25	*x* = 0.06474;<br>*y* = -0.01495;<br>*z* = 0.25	*x* = 0.6127(10);<br>*y* = 0.25;<br>*z* = -0.0505(12)	*x* = 0.5;<br>*y* = 0;<br>*z* = 0
O2	*x* = -0.2843(9);<br>*y* = 0.7872(9);<br>*z* = 0.4631(7)	*x* = 0.7137(14);<br>*y* = 0.7943(20);<br>*z* = 0.4613(9)	*x* = -0.28458;<br>*y* = 0.78294;<br>*z* = 0.46245	*x* = 0.788291;<br>*y* = 0.702938;<br>*z* = 0.025991	*x* = -0.28458;<br>*y* = 0.78294<br>*z* = 0.46245	*x* = 0.3051(8);<br>*y* = 0.0657(6);<br>*z* = 0.3099(8)	
Occupancy							
Sr	1	1	1	1	1	Ca: 1	Ba: 1
Zr	1	0.9	0.8	0.8	0.8	0.8	0.8
Y	/	0.1	0.2	0.2	0.2	0.2	0.2
O1	1	1	0.996	1	1	1	1
O2	1	1	0.966	1	1	1	
*U*iso (Å^2)							
Sr	0.00990(13)	0.01090(20)	0.0156(7)	0.0126(6)	0.0134(5)	Ca: 0.0026(3)	Ba: 0.00783(29)
Zr	0.00361(11)	0.00570(17)	0.0086(6)	0.0023(8)	0.0057(4)	0.01441(22)	0.0300(21)
Y	/	0.00570(17)	0.0086(6)	0.0023(8)	0.0057(4)	0.01441(22)	0.0300(21)
O1	0.0107(22)	0.0090(18)	0.0230(16)	0.050(26)	0.004(7)	0.018(5)	0.0443(22)
O2	0.0326(16)	0.0617(26)	0.0502(22)	0.046(5)	0.0538(18)	0.032(5)	/

$SrZr_{0.9}Y_{0.1}O_{3-\delta}$ (SZYO10) has 2% of the second phase SrY_2O_4 *Pnam*;

$SrZr_{0.8}Y_{0.2}O_{3-\delta}$ (SZYO20) has 8% of the second phase SrY_2O_4 *Pnam*;

SZYO20 after treated in H_2O or tested in wet air has 2% of the second phase $SrCO_3$ *Pmcn*;

$BaZr_{0.8}Y_{0.2}O_{3-\delta}$ (BZYO20) has 1% of the second phase Y_2O_3 *Ia-3*.

Supplementary Table 2. Atomic concentration of SZYO20 as on STEM-EDX analysis.

Samples	Atomic composition (%)					
	Sr	Zr	Y	O	Sr/(Zr+Y)	Zr/Y
SZYO20	20.65	15.93	4.60	58.82	1.006 (1)	3.46 (4)
Washed SZYO20	22.97	19.56	3.50	53.97	0.996	5.59

brackets indicate the expected ratio.

Supplementary Table 3. Summary of results of concentration cell experiments to identify the charge carrier in various electrolytes.

Electrolyte	ΔE(mV)	t_{Na+}	t_{OH-}
$CaZrO_3$ (CZO)	-85.352	0.148	0.852
$CaZr_{0.8}Y_{0.2}O_{3-\delta}$ (CZYO20)	-74.873	0.220	0.780
$SrZrO_3$ (SZO)	-63.002	0.244	0.756
$SrZr_{0.8}Y_{0.2}O_{3-\delta}$ (SZYO20)	-51.769	0.303	0.697
$BaZrO_3$ (BZO)	-43.9415	0.366	0.634
$BaZr_{0.8}Y_{0.2}O_{3-\delta}$ (BZYO20)	-42.278	0.384	0.616
Anion exchange membrane, (Fumapem FAA)	-104.40	0.073	0.927
Nafion 212 membrane	+76.69	0.872	0.128

All the perovskite oxide electrolyte pellets had been treated and washed in water at 90 °C to get rid of SrY_2O_4 or other residual soluble oxides before measured in a concentration cell.

Supplementary Table 4. Summary of conductivity and pH of the used water after conductivity measurement for pellets.

Oxide pellet	Temperature	Ionic conductivity measured of oxide pellets in water (μS cm^{-1})	Conductivity of water after conductivity measurement for pellets (μS cm^{-1})	pH of water after conductivity measurement for pellets
CZO	25 °C	173.80	47.93	7.63
CZYO20	22 °C	1010.17	109.4	9.72
SZO	21 °C	212.85	88.76	9.29
SZYO10	23 °C	1239.05	255.60	10.78
SZYO20	21 °C	2174.98	407.1	11.07
BZO	25 °C	34.10	41.82	7.69
BZYO20	22 °C	675.28	42.9	9.68

The conductivity and pH of the tested water were measured with meters immediately after the ionic conductivity measured via Solartron 1470/1455A frequency response analyser (FRA).

Supplementary Table 5. Comparison of OH^- conductivity with literature results.

Material	*T* (°C)	Test condition	Conductivity ($mS\ cm^{-1}$)	*E*a (eV)	Ref
Poly(ether-imide)-based AAEM	Room temperature	Water	2.28-3.51	~	[15]
$(LDH/QAPSF)_n$ film	Room temperature	Water	5.30-7.25	~	[16]
TEAOH–PAM-$mSiO_2$	Room temperature	75% RH	10	0.116	[17]
30% Qppo-PVA membrane	Room temperature	100% RH	151	0.154	[18]
QPAES/2.5% nano-ZrO_2 membrane	20	100% RH	10.1	0.176	[19]
PFB^+ thin film	25	95% RH	52	~	[20]
Quaternized poly ether imide membrane	30	Water	2.4	~	[21]
QPAF-4™ membrane	60	95% RH	64	~	[22]
Single-layer Mg-Al LDH nanosheet	60	80% RH	~100	0.35	[23]
PVA/PAADDA-i AAEM	60	Water	0.67	0.15-0.20	[24]
QP(DNB/NB-SiO2-25) membrane	80	1 M NaOH	9.33	~	[25]
A-PEI membrane	90	100% RH	6.7-44.2	0.25-0.30	[26]
SZYO20 pellet	30	Water	2.45	0.179±0.006	This work
SZYO20 pellet	70	100% RH	10.9 (OH^-/H^+ mix)	~	This work
CZYO20 pellet	80	1 M KOH	9.1-20.2	0.124±0.006	This work
CZYO20 pellet	90	6 M KOH	47.9-106.3	0.135±0.003	This work

Supplementary Table 6. Structural data for vacuum dried SZYO20 refined against room temperature NPD data.

Space group *Pbnm* (No. 62), a = 5.80895(13) Å, b = 5.82939(12) Å, c = 8.22087(20) Å, V = 278.380(5) Å^3.

Atom	***Wyckoff***	***x***	***y***	***z***	***Occ.***	**U_{iso} (Å^2)**
Sr1	4c	0.9926(8)	0.5201(3)	0.25	1[a]	0.0086(3)
Zr1	4a	0	0	0	0.86[b]	0.00315(24)[c]
Y1	4a	0	0	0	0.14[b]	0.00315(24)[c]
O1	4c	0.06474(7)	0.9851(5)	0.25	1[a]	0.0087[d]
O2	8d	0.71542(4)	0.78294(31)	0.46245 (26)	0.963(6)	0.0088(7)[e]

**ADP (Å^2)**	**U_{11}**	**U_{22}**	**U_{33}**	**U_{12}**	**U_{13}**	**U_{23}**
O1	0.0126(12)	0.0163(16)	0.0062(11)	-0.0031(22)	0	0

[a] Values refined close to 1 so were fixed to 1.
[b] Fixed Zr/Y occupancies based on refined oxygen vacancies.
[c] Fixed to have equivalent isotropic atomic displacement parameters.
[d] Equivalent isotropic atomic displacement parameters. Anisotropic displacement parameters (ADP) refined for the O1 site.
[e] Isotropic displacement parameters used for O2 site due to the correlation with oxygen vacancies.

Supplementary Table 7. Structural data for deuterated SZYO20 refined against room temperature NPD data.

Space group *Pbnm* (No. 62), *a* = 5.80866(14) Å, *b* = 5.82904(13) Å, *c* = 8.22026(22) Å, *V* = 278.329(10) Å^3.

Atom	***Wyckoff***	***x***	***y***	***z***	***Occ.***	U_{iso} **(Å^2)**
Sr1	4c	0.9936(6)	0.52644(27)	0.25	1[a]	0.00807(28)
Zr1	4a	0	0	0	0.86[b]	0.00284(19)[c]
Y1	4a	0	0	0	0.14[b]	0.00284(19)[c]
O1	4c	0.0650(6)	0.9843(4)	0.25	1[a]	0.0067[d]
O2	8d	0.71315(30)	0.78578(27)	0.46363(30)	1[a]	0.0134[d]
D1	8d	0.663(6)	0.158(6)	0.042(4)	0.07[e]	0.0708(12)

**ADP (Å^2)**	U_{11}	U_{22}	U_{33}	U_{12}	U_{13}	U_{23}
O1	0.0008(14)	0.0103(16)	0.0104(16)	-0.0116(21)	0	0
O2	0.0091(9)	0.0092(8)	0.0219(11)	-0.0062(15)	0.0116(20)	0.0038(16)

[a] Values refined close to 1 so were fixed to 1.
[b] Fixed perovskite Zr/Y occupancies based on the $Y(OH)_3$ phase fraction such that the total Zr/Y ratio for the sample remained 4:1.
[c] Fixed to have equivalent isotropic atomic displacement parameters.
[d] Equivalent isotropic atomic displacement parameters. Anisotropic displacement parameters (ADP) refined for each oxygen position.
[e] Value refined close to the expected occupancy for this site reflecting complete filling of the oxygen vacancies by OD groups. Hence for the final refinement the D occupancy was fixed.

Supplementary Table 8. Summary of second nearest neighbour O···O distances (in Å) and Zr-O-Zr bond angle (in degree) in selected undoped zirconate perovskite structures.

Structure	O-O (Å)	M-O-M (°)	Reference
$SrZrO_3$ (*Pbnm*)	3.478 / 3.579	156.7	[27]
$SrZrO_3$ (*I4/mcm*)	3.532 / 4.184	163.4	[28]
$CaZrO_3$ (*Pcmn*)	3.133 / 3.312	145.5	[29]
$BaZrO_3$ (*Pm3m*)	4.191 / 4.191	180.0	[29]

The Zr-O-Zr bond angle characterises the degree of octahedral tilting. Space groups are given in brackets for reference.

Supplementary Table 9. Summary of the relative densities of oxide pellets.

Oxide pellet sintered at different temperatures	Relative density of pellet	
	Real density calculated from pellet dimensions	Real density measured from Archimedes' Method
CZO-1300 °C	90%	96%
CZO-1500 °C	97%	99%
CZYO20-1300 °C	74%	93%
CZYO20-1500 °C	97%	99%
$SrZr_{1-x}Y_xO_{3-\delta}$ (x= 0, 0.1, 0.2)-1300 °C	65%	80-90%
SZYO20-1500 °C	79%	94%
BZO-1500 °C	75%	95%
BZYO20-1500 °C	46%	67%

Supplementary References

1 Takeguchi, T. *et al.* Layered perovskite oxide: a reversible air electrode for oxygen evolution/reduction in rechargeable metal-air batteries. *Journal of the American Chemical Society* **135**, 11125-11130 (2013).

2 Dai, H.-Y., Yang, H.-M., Jian, X., Liu, X. & Liang, Z.-H. Performance of Ag2O/Ag Electrode as Cathodic Electron Acceptor in Microbial Fuel Cell. *Acta Metallurgica Sinica (English Letters)* **30**, 1243-1248, doi:10.1007/s40195-017-0616-1 (2017).

3 Takahashi, H. *et al.* Ion conduction in layered oxide solid electrolyte. *ECS Transactions* **33**, 1861 (2010).

4 Watanabe, H., Takahashi, H., Takeguchi, T., Yamanaka, T. & Ueda, W. Performance of solid alkaline fuel cells employing layered perovskite-type oxides as electrolyte. *ECS Transactions* **33**, 1825 (2010).

5 Takeguchi, T. *et al.* Layered perovskite oxide: a reversible air electrode for oxygen evolution/reduction in rechargeable metal-air batteries. *J Am Chem Soc* **135**, 11125-11130, doi:10.1021/ja403476v (2013).

6 Slodczyk, A., Colomban, P., Upasen, S., Grasset, F. & André, G. Structural stability of anhydrous proton conducting SrZr0. 9Er0. 1O3− δ perovskite ceramic vs. protonation/deprotonation cycling: Neutron diffraction and Raman studies. *Journal of Physics and Chemistry of Solids* **83**, 85-95 (2015).

7 Colomban, P., Tran, C., Zaafrani, O. & Slodczyk, A. Aqua oxyhydroxycarbonate second phases at the surface of Ba/Sr - based proton conducting perovskites: a source of confusion in the understanding of proton conduction. *Journal of Raman Spectroscopy* **44**, 312-320 (2013).

8 Shkerin, S. *et al.* Raman spectroscopy of SrZrO3 based proton conducting electrolyte: Effect of Y-doping and Sr-nonstoichiometry. *International Journal of Hydrogen Energy* **46**, 17007-17018 (2021).

9 Sears, V. F. Neutron scattering lengths and cross sections. *Neutron News* **3**, 26-37, doi:10.1080/10448639208218770 (1992).

10 Bai, S., Perevoshchikova, N., Sha, Y. & Wu, X. The effects of selective laser melting process parameters on relative density of the AlSi10Mg parts and suitable procedures of the archimedes method. *Applied Sciences* **9**, 583 (2019).

11 Draksharapu, A. *Shedding light on active species in Fe, Ni and Cu catalysis: photochemical, spectroscopic and electrochemical studies.* Doctor of Philosophy thesis, University of Groningen, (2013).

12 Huang, G., Zhanglian, H., Shizhu, Z., Pengyue, Z. & Xianping, F. Synthesis of yttrium oxide nanocrystal via solvothermal process. *Journal of rare earths* **24**, 47-50 (2006).

13 Aghazadeh, M., Ghaemi, M., Nozad Golikand, A., Yousefi, T. & Jangju, E. Yttrium oxide nanoparticles prepared by heat treatment of cathodically grown yttrium hydroxide. *International Scholarly Research Notices* **2011** (2011).

14 Irvine, J. T., Sinclair, D. C. & West, A. R. Electroceramics: characterization by impedance spectroscopy. *Advanced materials* **2**, 132-138 (1990).

15 Wang, G., Weng, Y., Chu, D., Xie, D. & Chen, R. Preparation of alkaline anion exchange membranes based on functional poly (ether-imide) polymers for potential fuel cell applications. *Journal of Membrane Science* **326**, 4-8 (2009).

16 Xu, X. *et al.* Hydroxide-ion-conductive gas barrier films based on layered double hydroxide/polysulfone multilayers. *Chemical communications* **54**, 7778-7781 (2018).

17 Li, J. & Lian, K. The effect of SiO 2 additives on solid hydroxide ion-conducting polymer electrolytes: A Raman microscopy study. *Physical Chemistry Chemical Physics* **20**, 7148-7155 (2018).

18 Zhang, K., McDonald, M. B., Genina, I. E. & Hammond, P. T. A highly conductive and mechanically robust OH–conducting membrane for alkaline water electrolysis. *Chemistry of Materials* **30**, 6420-6430 (2018).

19 Li, X., Yu, Y. & Meng, Y. Novel quaternized poly (arylene ether sulfone)/nano-ZrO2 composite anion exchange membranes for alkaline fuel cells. *ACS applied materials & interfaces* **5**, 1414-1422 (2013).

20 Wang, F., Wang, D. & Nagao, Y. OH− Conductive Properties and Water Uptake of Anion Exchange Thin Films. *ChemSusChem* **14**, 2694-2697 (2021).

21 Elangovan, M. & Dharmalingam, S. Preparation and performance evaluation of poly (ether-imide) based anion exchange polymer membrane electrolyte for microbial fuel cell. *International Journal of Hydrogen Energy* **41**, 8595-8605 (2016).

22 Zhegur-Khais, A., Kubannek, F., Krewer, U. & Dekel, D. R. Measuring the true hydroxide conductivity of anion exchange membranes. *Journal of Membrane Science* **612**, 118461 (2020).

23 Sun, P. *et al.* Single-layer nanosheets with exceptionally high and anisotropic hydroxyl ion conductivity. *Science advances* **3**, e1602629 (2017).

24 Qiao, J., Fu, J., Liu, L., Liu, Y. & Sheng, J. Highly stable hydroxyl anion conducting membranes poly (vinyl alcohol)/poly (acrylamide-co-diallyldimethylammonium chloride)(PVA/PAADDA) for alkaline fuel cells: Effect of cross-linking. *International journal of hydrogen energy* **37**, 4580-4589 (2012).

25 He, X. *et al.* Crosslinked hydroxyl ‐ conductive copolymer/silica composite membranes based on addition ‐ type polynorbornene for alkaline anion exchange membrane fuel cell applications. *Polymer Engineering & Science* **58**, 13-21 (2018).

26 Oh, B. H., Kim, A. R. & Yoo, D. J. Profile of extended chemical stability and mechanical integrity and high hydroxide ion conductivity of poly (ether imide) based membranes for anion exchange membrane fuel cells. *International Journal of Hydrogen Energy* **44**, 4281-4292 (2019).

27 Knight, K. S. & Bull, C. L. Low temperature and high pressure thermoelastic and crystallographic properties of SrZrO3 perovskite in the Pbnm phase. *Solid State Sciences* **62**, 90-104 (2016).

28 Kennedy, B. J., Howard, C. J. & Chakoumakos, B. C. High-temperature phase transitions in SrZrO 3. *Physical Review B* **59**, 4023 (1999).

29 Levin, I., Amos, T. G., Bell, S. M. & Farber, L. T. a. Vanderah, RS Roth and BH Toby, Phase Equilibria, Crystal Structures, and Dielectric Anomaly in the BaZrO3–CaZrO3 System. *J. Solid State Chem* **175**, 170-181 (2003).
